# The genetic basis of apple shape and size unraveled by digital phenotyping

**DOI:** 10.1093/g3journal/jkae045

**Published:** 2024-03-05

**Authors:** Beat Keller, Michaela Jung, Simone Bühlmann-Schütz, Marius Hodel, Bruno Studer, Giovanni A L Broggini, Andrea Patocchi

**Affiliations:** Division of Plant Breeding, Agroscope, Mueller-Thurgau-Strasse 29, Waedenswil 8820, Switzerland; Division of Plant Breeding, Agroscope, Mueller-Thurgau-Strasse 29, Waedenswil 8820, Switzerland; Molecular Plant Breeding, Institute of Agricultural Sciences, ETH Zurich, Universitaetstrasse 2, Zurich 8092, Switzerland; Division of Plant Breeding, Agroscope, Mueller-Thurgau-Strasse 29, Waedenswil 8820, Switzerland; Division of Plant Breeding, Agroscope, Mueller-Thurgau-Strasse 29, Waedenswil 8820, Switzerland; Molecular Plant Breeding, Institute of Agricultural Sciences, ETH Zurich, Universitaetstrasse 2, Zurich 8092, Switzerland; Molecular Plant Breeding, Institute of Agricultural Sciences, ETH Zurich, Universitaetstrasse 2, Zurich 8092, Switzerland; Division of Plant Breeding, Agroscope, Mueller-Thurgau-Strasse 29, Waedenswil 8820, Switzerland

**Keywords:** *Malus × domestica*, GWAS, genomics-informed breeding

## Abstract

Great diversity of shape, size, and skin color is observed among the fruits of different apple genotypes. These traits are critical for consumers and therefore interesting targets for breeding new apple varieties. However, they are difficult to phenotype and their genetic basis, especially for fruit shape and ground color, is largely unknown. We used the FruitPhenoBox to digitally phenotype 525 genotypes of the apple reference population (apple REFPOP) genotyped for 303,148 single nucleotide polymorphism (SNP) markers. From the apple images, 573 highly heritable features describing fruit shape and size as well as 17 highly heritable features for fruit skin color were extracted to explore genotype–phenotype relationships. Out of these features, seven principal components (PCs) and 16 features with the Pearson’s correlation *r* < 0.75 (selected features) were chosen to carry out genome-wide association studies (GWAS) for fruit shape and size. Four PCs and eight selected features were used in GWAS for fruit skin color. In total, 69 SNPs scattered over all 17 apple chromosomes were significantly associated with round, conical, cylindrical, or symmetric fruit shapes and fruit size. Novel associations with major effect on round or conical fruit shapes and fruit size were identified on chromosomes 1 and 2. Additionally, 16 SNPs associated with PCs and selected features related to red overcolor as well as green and yellow ground color were found on eight chromosomes. The identified associations can be used to advance marker-assisted selection in apple fruit breeding to systematically select for desired fruit appearance.

## Introduction

Apple (*Malus* × *domestica* Borkh.) cultivars inherited their great diversity of fruit qualities and sizes from the wild ancestors (Cornille *et al*. 2014). During domestication, the apple fruits have increased in size, and they have been selected for specific appearance (e.g. red skin color), increasingly reflecting human consumer preferences (Cornille *et al*. 2014; Duan *et al*. 2017; Migicovsky *et al*. 2021). Today’s cultivated apple is the third most produced fruit crop in the world (http://www.fao.org/faostat). Its fruit appearance, that can be described as the external fruit quality including traits such as fruit shape, size, and skin color, has been found to be a primary purchase-driving criterion for consumers (Kays 1999; Musacchi and Serra 2018).

To create varieties with specific fruit size, information about genetic regions controlling the trait can benefit apple breeding. Genes potentially contributing to the increase in fruit size during apple speciation before its domestication associated with fruit size on chromosome (Chr) 11 and 16 have been identified (Yao *et al*. 2015; Duan *et al*. 2017). Additionally, a locus on Chr 15 was associated with fruit vertical diameter of wild and cultivated *Malus* accessions (Liao *et al*. 2021), and two loci on Chr 8 and 15 were found to be significantly associated with fruit weight in *M.* × *domestica* (Devoghalaere *et al*. 2012). In the same two genetic regions, selective sweeps in *M.* × *domestica* compared with *M. sieversii* and *M. sylvestris* were reported (Duan *et al*. 2017). The genetic basis of fruit size in *M.* × *domestica* has been further studied on traits such as fruit weight, diameter, and length reporting many quantitative trait loci (QTL) across all apple chromosomes (Kenis *et al*. 2008; Devoghalaere *et al*. 2012; Chang *et al*. 2014; Costa 2015; Liu *et al*. 2016; Minamikawa *et al*. 2021). Despite the high abundance of known associations with traits related to fruit size, additional discovery, validation, and consolidation of the identified associations could facilitate future application in genomics-assisted breeding.

The quantitative inheritance of fruit shape in apple was postulated already in 1960 (Brown 1960). Fruit shape as a subjective score of fruit roundness appeared under strong genetic control (Hardner *et al*. 2016). Description of fruit shape as a ratio between fruit length and diameter has led to discovery of QTL for length/diameter on multiple linkage groups (Sun *et al*. 2012; Chang *et al*. 2014). In tomato, various phenotypic measurements of fruit morphology based on fruit boundaries (contours) automatically extracted using the Tomato Analyzer have resulted in the detection of several QTL for fruit shape and size (Brewer *et al*. 2006; Gonzalo *et al*. 2009). The Tomato Analyzer approach was recently applied to measure fruit sections for 355 genotypes of the apple reference population (apple REFPOP), leading to a discovery of numerous marker-trait associations for shape and size (Dujak and Aranzana 2023). Applying a similar, nondestructive digital phenotyping approach to a larger number of genotypes may help further elucidate the genetic background of apple fruit shape and size.

Just like fruit shape and size, skin color is important in determining the esthetic appeal of fruits and overall consumer preference for apples. Apples typically have a ground color, which is the primary background color of the fruit, and an overcolor, which represents an additional layer of red or yellow color that overlays the ground color. The causal loci for red fruit skin color in apples have been broadly studied in the past. Several transcription factors control anthocyanin production in the fruit skin during ripening (Takos *et al*. 2006; Espley *et al*. 2007). A major QTL for red overcolor has been repeatedly reported on Chr 9 at around 30 Mbp (Chagné *et al*. 2016; Duan *et al*. 2017; Moriya *et al*. 2017). However, the genomic control of green and yellow fruit skin color was rarely studied in apple. Visually assessed ground color, a gradient between green and yellow fruit skin color, was associated with QTL distributed over 13 chromosomes (Jung *et al*. 2022). Jung *et al*. (2022) further demonstrated that green color estimated digitally using an automated fruit sorting machine was an inverse of the digitally and visually estimated red overcolor, and all these skin color traits were associated with the same single nucleotide polymorphism (SNP) marker on Chr 9 at 33.8 Mbp. Furthermore, SNPs of minor effects associated with green color were identified on Chr 6, 10, and 17, none of the SNPs associated with green color overlapped with the SNPs reported for ground color, and no SNPs were reported for yellow color (Jung *et al*. 2022). Additional phenotypic information for green and yellow skin color may allow for a more detailed description and the advancement of biological understanding of these traits.

Digital phenotyping is increasingly used in fruit trees to ease quantification of traits (Huang *et al*. 2020). In apple trees, RGB images have been used to estimate tree architecture or count fruits on trees in the orchard (Zine-El-Abidine *et al*. 2021; Zhang *et al*. 2023). The recently developed phenotyping device FruitPhenoBox, an RGB camera system combined with automatized image analysis, showed as a low-cost tool suitable for extraction of fruit features (Kirchgessner *et al*. 2023). However, the application of fruit imaging with the FruitPhenoBox for the assessment of fruit shape, size, and skin color with the aim of identifying genetic associations with these traits remained to be tested.

To dissect the genetic basis of apple fruit shape, size, and skin color, this study used the FruitPhenoBox (Kirchgessner *et al*. 2023) to digitally phenotype the apple REFPOP, a diverse apple reference population located at Agroscope in Waedenswil, Switzerland (Jung *et al*. 2020). We aimed to (1) extract new phenotypic features from images taken with the FruitPhenoBox, (2) compare the features with apple fruit size and skin color traits obtained visually and using a commercial sorting machine, (3) perform genome-wide association studies (GWAS) on heritable features, and (4) identify genetic markers and multilocus genotypes that could be used for genomics-assisted breeding towards the improvement of apple fruit appearance.

## Materials and methods

### Plant material and phenotyping

The apple REFPOP, which consisted of 269 accessions and 265 progeny, was planted in 2016 in Waedenswil, Switzerland (Jung *et al*. 2020). The fruits of the apple REFPOP were used to study fruit shape, size, and skin color. The genotypes were replicated at least twice in a complete randomized block design. All fruits of each genotype replicate (tree) were manually harvested. Harvest date was defined for each tree separately as the date when more than 50% of all fruits reached their full physiological maturity (Jung *et al*. 2020). The FruitPhenoBox (Kirchgessner *et al*. 2023) was used to phenotype 175 and 506 genotypes harvested in 2019 and 2020, respectively. Of these genotypes, 156 genotypes were phenotyped in both years. The phenotyping was performed on harvest date at the level of individual trees. When more than five fruits per tree were available, a random sample of five representative fruits was chosen for imaging. When five or fewer fruits were produced by a tree, all available fruits were imaged. Individual fruits with shape or size clearly different from the rest of the group, or diseased, deformed, or damaged fruits, were excluded before sampling and imaging.

To compare measurements obtained using the FruitPhenoBox with different measurement approaches, a set of visually scored traits and traits measured by a commercial sorting machine (GREEFA iQS4 v.1.0) in 2019 and 2020 available from Jung *et al*. (2022) were included in the study as supplementary traits. The visually scored supplementary traits ground color (labeled as Color_ground) and percentage of red overcolor (Color_over) were measured as described by Jung *et al*. (2022). The supplementary traits measured by the sorting machine were comprised of the percentage of green (labeled as Green), yellow (Yellow), and red fruit skin color (Red), fruit diameter (Diameter), length (Length), maximum size (maxSize), volume (Volume), and single fruit weight (Fruit_weight_single). For each of the supplementary traits measured by the sorting machine, the values were averaged across all fruits produced by a tree. From all produced fruits, a random subset of 20 fruits (or at least five fruits in case 20 fruits were not available) was chosen for visual scoring. The visually scored subset was used to select fruits for phenotyping with the FruitPhenoBox as described above.

### Genotyping

The plant material was accompanied by a comprehensive genome-wide SNP marker dataset. In summary, SNPs were obtained from two partially overlapping SNP arrays with varying resolutions, namely, (1) the Illumina Infinium 20 K SNP genotyping array (Bianco *et al*. 2014) and (2) the Affymetrix Axiom Apple 480 K SNP genotyping array (Bianco *et al*. 2016). The SNPs were curated as described by Jung *et al*. (2020). Subsequently, these datasets were merged using imputation with Beagle 4.0 (Browning and Browning 2007) utilizing pedigrees (Muranty *et al*. 2020). Nonpolymorphic markers were eliminated, resulting in a final set of 303,148 biallelic SNPs. The SNP positions were determined based on the apple reference genome derived from the doubled-haploid GDDH13 (v1.1) (Daccord *et al*. 2017).

### Imaging with the FruitPhenoBox

**Fig. 1.**
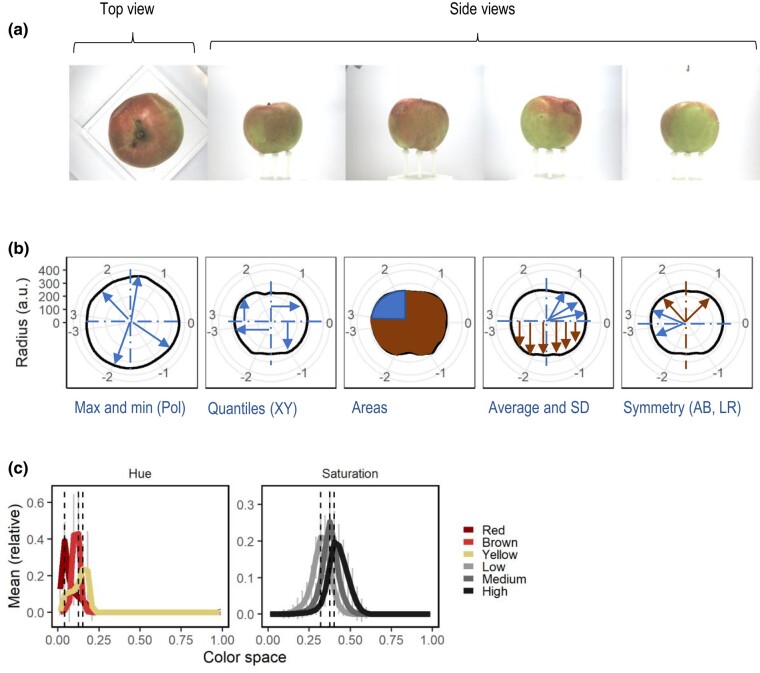
Example of feature definition with the FruitPhenoBox. a) One top and four side view RGB images were taken for each fruit. b) For shape and size feature definition, fruit contour was derived in polar (Pol) and Cartesian (XY) coordinates for each fruit and image, displayed here as radius in absolute values (a.u.). Features such as maximum and minimum coordinate values (Max and min), quantiles, areas, averages, and standard deviations (SD) as well as symmetries were extracted either from the full fruit contour or the top (A), bottom (B), left (L), or right (R) fruit sections or their combinations. c) For the color features, histograms were derived for hue and saturation color spaces. Color features were extracted at the positions of peaks obtained for hierarchically clustered genotypes.

The FruitPhenoBox, its imaging procedure, and image segmentation were described by Kirchgessner *et al*. (2023). Briefly, the FruitPhenoBox consisted of five RGB cameras and a scale. Four cameras were placed in the corners of the FruitPhenoBox, and one camera was positioned above the fruit (Fig. 1a). For imaging, a single fruit was placed on a platform on the scale. By a manual command, the weight was measured, and one image of the fruit was taken by each of the five cameras. The background of each image was identified and removed using image segmentation implemented in MATLAB (MATLAB 2010) as described by Kirchgessner *et al*. (2023). The apple contour was derived in Cartesian and polar coordinates. The polar coordinates were expressed as radius and polar angle for 360 points, one point for each degree. The Cartesian coordinates were expressed as 360 corresponding x and y values. The color of the segmented fruit was derived as hue and saturation values ranging from 0 to 1 representing 36 averaged values among the color space over all segmented pixels.

Apple fruits whose dimension on the side-view images was smaller than 150 pixels were not further considered because they did not fully cover the opposite camera lens and caused the image segmentation to fail. To fully remove the apple stalk in the apple side-view images, data points above 1.5 times the interquartile range (the difference between the first and the third quartile) of the upper quarter of the contour data were removed.

### Feature extraction for shape and size

From the apple fruit contours obtained from FruitPhenoBox, 1D features for shape and size were systematically processed (Table 1, Fig. 1b). For the points on the fruit contour defined in polar coordinates (Pol), the distance of the point from the center of the coordinate system, i.e. the radius, was used in feature extraction. The points in the Cartesian coordinate system were expressed as individual coordinates for both axes (X measuring fruit diameter and Y measuring fruit length). Different fruit sections were determined as the top (A), bottom (B), left (L), or right (R) half of the fruit, as well as intersections of the fruit halves, i.e. fruit quarters (A.L, A.R, B.L, B.R) and unions of the halves, i.e. the full fruit (AB, LR). Points from the fruit contour derived in Cartesian and polar coordinates within a chosen fruit section were used to calculate different types of features, namely the mean (Av), the standard deviation (SD), the minimum (Min), or maximum (Max) value, the 0.25, 0.50, or 0.75 quartile (Q25, Q50, Q75), the area, the ratio between Max and Min value (RatM), and the Max value relative to the Av value (MaxR). The area was calculated by summing up 1°-radius increments in the specified section. For each type of feature, the absolute value (Abs) was taken from the features extracted on different sections. For the opposite fruit halves, absolute values were first extracted and then the ratio (Rat) and the sum (Sum) were calculated across the fruit halves. The ratio or the sum of top vs bottom halves was calculated as average (Rat.Av and Sum.Av) and SD (Rat.SD and Sum.SD). Hereby, the top and bottom halves were calculated from quarter values using the corresponding left–right fruit quarters (AB.LR). Additionally, symmetry between L and R as well as A and B was calculated as the Av or SD of the difference between corresponding absolute values of radius of each fruit half (Sym.abs, sections LR, and AB). Similarly, the symmetry measure Sym.abs was obtained for individual apple sections A, B, L, and R when comparing the opposite quarters within the fruit halves. Additionally, a relative symmetry (Sym.rel) was derived for the same apple sections as Sym.abs, but the measure Sym.rel was normalized for the area of the apple section.

**Table 1.** Feature definition key.

Tree-level	Fruit-level	Data type	Calculation	Section	Feature type	Selected feature
Mean (Av)	Top	Pol	Abs	A.L	Av	
SD	Side average (SAv)			A.R	SD	Av_SSD_Pol_Abs_B.L_SD
	Side SD (SSD)			B.L	Max	
				B.R	Min	
					Q25	
					Q50	
					Q75	
					Area	
					RatM	
Mean (Av)	Top	Pol	Av	A.LR	Av	
SD	Side average (SAv)		SD	B.LR	SD	
	Side SD (SSD)				Max	Av_SSD_Pol_SD_B.LR_Max
					Min	Av_SAv_Pol_SD_A.LR_Min
					Q25	Av_SSD_Pol_SD_B.LR_Q25
					Q50	
					Q75	
					Area	
					RatM	Av_Top_Pol_Av_A.LR_RatM
Mean (Av)	Top	Pol	Abs	AB	Av	
SD	Side average (SAv)		Sum.SD	AB.LR	SD	Av_SSD_Pol_Rat.Av_AB.LR_SD
	Side SD (SSD)		Sum.Av		Max	Av_SSD_Pol_Rat.Av_AB.LR_Max
			Rat.SD		Min	Av_SSD_Pol_Rat.Av_AB.LR_Min
			Rat.Av		Q25	
					Q50	Av_SAv_Pol_Rat.Av_AB.LR_Q50
					Q75	
					Area	
					RatM	Av_SSD_Pol_Abs_AB_RatM Av_SAv_Pol_Sum.SD_AB.LR_RatM
Mean (Av)	Top	Pol	Av	LR	Sym.rel	
SD	Side average (SAv)		SD	AB	Sym.abs	
	Side SD (SSD)					
Mean (Av)	Top	Pol	Av	A	Sym.rel	Av_SSD_Pol_SD_R_Sym.rel
SD	Side average (SAv)		SD	B		Av_SAv_Pol_SD_B_Sym.rel
	Side SD (SSD)			L	Sym.abs	
				R		
Mean (Av)	Top	X	Abs	A	Av	
SD	Side average (SAv)	Y		B	SD	
	Side SD (SSD)				Max	
		XY	Sum	A	Min	
			Rat	B	Q25	Av_SSD_X_Abs_A_Q25 Av_SSD_X_Abs_B_Q25
					Q50	
					Q75	
					RatM	
					MaxR	Av_SAv_XY_Sum_A_MaxR
Mean (Av)	Side average (SAv)	Hue	Abs	AB	Red	SD_SAv_Hue_Abs_AB_Red
SD	Side SD (SSD)				Yellow	Av_SSD_Hue_Abs_AB_Yellow SD_SAv_Hue_Abs_AB_Yellow
					Brown	Av_SSD_Hue_Abs_AB_Brown Av_SAv_Hue_Abs_AB_Brown
		Sat	Abs	AB	Low	SD_SSD_Sat_Abs_AB_Low
					Medium	Av_SAv_Sat_Abs_AB_Medium
					High	Av_SAv_Sat_Abs_AB_High

Names of the extracted features represent combinations of the different row entries over the columns from left to right, with the names of the selected features displayed in the rightmost column. For the shape and size feature definition, the FruitPhenoBox was used to extract fruit contours from images of individual apples. Mean (Av) or standard deviation (SD) was calculated over up to five apples per tree that were imaged with one camera from the top view (Top) and four side-view cameras. The four side-view images were either averaged (side average, SAv) or their SD was obtained (side SD, SSD). The apple contour was defined by points expressed in polar (Pol) or Cartesian coordinates. The points in the Cartesian coordinate system were defined as individual coordinates for two axes (X measuring fruit diameter, Y measuring fruit length). Different fruit sections were determined as the top (A), bottom (B), left (L), or right (R) half of the fruit, fruit quarters (A.L, A.R, B.L, B.R) and full fruits (AB, LR). Points from the fruit contour within a chosen fruit section were used to calculate different types of features, namely the mean (Av), the standard deviation (SD), the minimum (Min), or maximum (Max) value, the 0.25, 0.50, or 0.75 quartile (Q25, Q50, Q75), the area, the ratio between Max and Min value (RatM), the Max value relative to the Av value (MaxR) and the symmetry features (Sym.abs, Sym.res). To calculate each type of feature, the absolute value (Abs) was taken from the features extracted on different fruit sections, or the absolute values were first extracted for the opposite fruit halves, and then the ratio (Rat) and the sum (Sum) were calculated across the fruit halves. The ratio or the sum of top vs bottom halves was calculated as average (Rat.Av, Sum.Av) and SD (Rat.SD, Sum.SD). Hereby, the top and bottom halves were calculated from quarter values using the corresponding left–right fruit quarters (AB.LR). For the color features, histograms for hue (Hue) and saturation (Sat) color spaces were used to define features for red, brown, and yellow hue as well as low, medium, and high saturation.

One image from top view (Top) and four images from side view (Side) were taken for each fruit. The features were corrected for camera effects in order to remove potential technical error due to the camera settings and position. Phenotypes (*y_ijkl_*) of the *i*th genotype, the *j*th year, and the *k*th replicate (tree) taken by the *l*th camera were corrected with a fixed effect for the camera (*C_l_*_(*j*)_) nested in year using the model:


(1) $$y_{ijkl}=C_{l\left(j\right)}+\varepsilon_{ijkl}.$$

The residuals (*ɛ_ijkl_*) were then used as features (*y_ijkl_*) for further analysis. For the four side-view images of a fruit, the feature values were averaged (SAv) or their standard deviation (SSD) was calculated. Mean values (Av) and standard deviations (SD) were calculated across up to five fruits harvested from each tree (replicate of a genotype). For each feature, the feature extraction finally resulted in one value per tree and year, i.e. one value for each combination of genotype, replicate, and year.

### Feature extraction for skin color

From the color histogram of each image with its background removed, features for color were extracted (Table 1). Initially, a histogram for either hue or saturation color space was obtained for each genotype by averaging all images from side view taken for the genotype. The average histograms per genotype were hierarchically clustered and three clusters of genotypes were defined for each color space. Average histogram was plotted for each of the three clusters of genotypes and the two color spaces, and the maximum values of the histograms (peaks) were identified (Fig. 1c). Next, the feature values at the peaks (peak ± 0.05 of the color space) were extracted for each fruit image separately. They were categorized into red, brown, and yellow hue as well as low, medium, and high saturation. The green colored apple fruits were not sufficiently represented to form an own peak.

The features were corrected for the camera effect using model 1. For all five images of a fruit, the color feature values were averaged (SAv) or their standard deviation (SSD) was calculated. Then, the mean values (Av) and standard deviations (SD) across up to five fruits harvested from each tree (replicate of a genotype) were calculated. Color features were always based on the full fruit (AB). For each feature, the feature extraction finally resulted in one value for each combination of genotype, replicate, and year.

### Statistical analysis

For the extracted features, outlier data points were removed when they were 15 times larger or lower than the size of the interquartile range. For every feature, adjusted means of each genotype (*G_i_*) were modeled for each extracted feature value per tree (*y_ijk_*) as the following:


(2) $$y_{ijk}=G_{i}+Y_{j}+R_{k(j)}+\varepsilon_{ijk},$$

where *Y_j_* was a fixed effect for the year, *R_k_*_(*j*)_ was a fixed effect for the replicated genotype nested in year, and *ɛ_ijk_* was the error term. For these adjusted features, clonal mean heritability (*H*^2^) was calculated as the genotypic variance ($\sigma_{g}^{2}$) divided by the sum of genotype and the error variance ($\sigma_{\varepsilon }^{2}$) adjusted for the average number of replicates over both years (${\bar{n}}_{r}$):


(3) $$H^{2}=\frac{\sigma_{g}^{2}}{\sigma_{g}^{2}+(\sigma_{\varepsilon }^{2}/{\bar{n}}_{r})}.$$

To reduce dimensions of the set of adjusted features, principal component analysis (PCA) was carried out. The PCA was performed separately for (1) the fruit shape and size features as well as for (2) fruit skin color features using the R package FactoMineR (Lê *et al*. 2008). The adjusted features showing an *H*^2^ lower than 0.4 were removed prior to PCA to focus the analysis on highly heritable features. From the highly heritable features with an *H*^2^ higher than 0.6, a set of features with a Pearson correlation lower than 0.75 between each other (selected features) was chosen using the *findCorrelation* command of the R package caret (Kuhn 2008). These selected features and supplementary traits measured by the conventional methods (sorting machine and visual scoring) were correlated to the principal components (PCs) and overlayed on a biplot.

The GWAS was carried out using the Bayesian-information and Linkage-disequilibrium Iteratively Nested Keyway (BLINK) algorithm (Huang *et al*. 2018). BLINK implemented a process for selecting associations based on linkage-disequilibrium information while using SNPs as covariates and controlling for population structure. The number of PCs (derived from the highly heritable features) to be used for GWAS was defined so that approx. 80% of the total variance was explained by the retained PCs. The GWAS was performed for the first seven PCs (PC1–PC7) for fruit shape and size, first four PCs for fruit skin color, and for the set of selected features for fruit shape, size, and color. For GWAS, the imputed SNP matrix for the apple REFPOP genotypes was obtained from Jung *et al*. (2020), and the SNPs were filtered for minor allele frequency of 0.05. The position of the SNP markers was determined according to the reference genome GDDH13 (v1.1) (Daccord *et al*. 2017). The first three PCs of the SNP matrix were used to correct for population structure in GWAS, mainly to adjust for the genetic differences between progeny and accessions. The *P-*values resulting from GWAS were Bonferroni-corrected with the significance threshold set to the 5% level, or (where indicated) to the 1% level for in-depth analysis. To assess the additive effect of all SNPs associated with one shape and size PC, or all associations with PCs and selected features, the SNPs ordered by a decreasing −log_10_(*P*) value were included in a linear regression model, and the regression sum of squares explained by each SNP was estimated.

To assess the combined effects of associated SNPs in a multilocus genotype analysis, groups of associated SNPs were created for (1) each of the first five shape and size PCs based on all SNPs associated with the PCs, (2) the shape and size PCs and selected features from associated SNPs located within 0.1 Mbp distance on the same chromosome (further denoted as groups of physically linked SNPs), (3) the selected feature for symmetry (Av_SSD_Pol_SD_R_Sym.rel), and (4) the selected feature for color (SD_Sav_Hue_Abs_AB_Yellow). The associated SNPs were ordered according to their position on the reference genome GDDH13 (v1.1) (Daccord *et al*. 2017). Then, separately for each SNP group, the allele dosages (0, 1, or 2 alternative alleles) of the associated SNPs were collapsed into combinations of allele dosages for each genotype. These combinations of allele dosages were called multilocus genotypes. The number of digits in multilocus genotypes varied depending on the number of SNPs within each group of associated SNPs. The number of multilocus genotypes for each group of associated SNPs was equal to the number of all available combinations of allele dosages found among the studied genotypes. For each multilocus genotype based on groups of physically linked SNPs, the average side-view fruit contour was plotted in absolute and relative values (mean = 0 and standard deviation = 1).

Major effects of the SNPs associated with fruit shape and size PCs and selected features were assessed from the visualization of the average side-view fruit contour for each allele dosage (0, 1, or 2 alternative alleles). The associated SNPs were classified as affecting fruit size (size), leading to lengthened fruits (conical shape) or very long fruits (cylindrical shape). The standard fruit shape for all classes was round, with the opposite effect of one of the alleles in a SNP resulting in conical or cylindrical shape (designated as nonstandard allele). For fruit size, the allele resulting in enlarged fruit contours was designated as nonstandard. Following these principles, the major effects were also visually assessed for the average side-view fruit contours for multilocus genotype clusters and multilocus genotypes based on groups of physically linked SNPs associated with PCs and selected features for fruit shape and size. For the fruit skin color PCs and selected features, the major effects of allele dosage (0, 1, or 2 alternative alleles) were visually assessed from feature distributions along the hue and saturation spectra. Allele associated with a peak in the red part of the hue spectrum was assumed as the standard, with the opposite allele classified as being associated with light green or yellow hue. Alleles associated with light green or yellow hue were designated nonstandard.

All statistical analyses and data formatting in this article were performed with R (R Core Team 2022).

### Comparison with previously published QTL

Earlier reports on QTL mapping and GWAS in apple that were extensively reviewed and reported by Jung *et al*. (2022) were taken for comparison with the associations reported in this study. From the review of 41 studies, the trait group named fruit size was considered for the comparison with the SNPs associated with fruit shape and size PCs and selected features, and the trait groups fruit ground color and overcolor were considered for the comparison with the associations with the fruit color PCs and selected features. Jung *et al*. (2022) visually assigned the positions of the reviewed associations within respective chromosomes to the three chromosome segments, i.e. top, center, and bottom. To compare chromosome-segment combinations found for loci in our and the earlier studies, the SNPs on each of the 17 chromosomes in our dataset were categorized into three equally sized chromosome segments, while the length of each chromosome was defined by its last marker. Following these principles, QTL obtained for the ratio between fruit length and diameter found by Sun *et al*. (2012) and Chang *et al*. (2014), and results of the study of fruit size by Liao *et al*. (2021), which were not included in the review of Jung *et al*. (2022), were added to the comparison with shape and size PCs and selected features.

Associations with fruit shape and size as well as color traits reported recently by Duan *et al*. (2017), Minamikawa *et al*. (2021), Liao *et al*. (2021), Jung *et al*. (2022), and Dujak and Aranzana (2023) were compared with the results obtained in our study. Position of DNA sequences containing selective sweeps associated with fruit size published by Duan *et al*. (2017) were estimated by the alignment of the sequences to the reference genome of the ‘Golden Delicious’ doubled-haploid line GDDH13 (v1.1) (Daccord *et al*. 2017) using the Genome Database for Rosaceae (https://www.rosaceae.org). Previously published molecular markers and significant SNPs identified in the current study were considered as co-localized when they were less than 0.1 Mbp distant from each other.

## Results

### Shape and size features

**Fig. 2.**
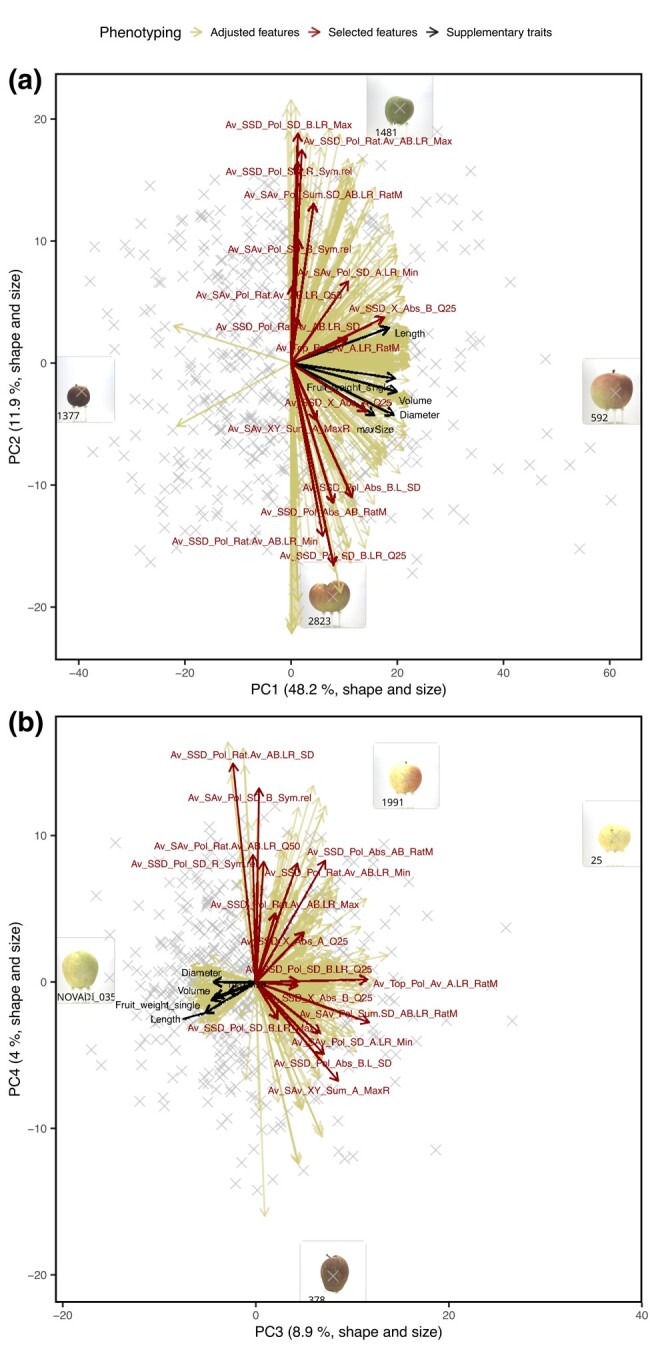
Principal component (PC) analysis of highly heritable features for fruit shape and size. The selected features for fruit shape and size and supplementary features measured using the sorting machine were overlayed in a biplot. Images of extreme genotypes of the apple REFPOP were shown in boxes and labeled with their respective genotype codes. a) PC1 and PC2, b) PC3 and PC4.

A total of 1,248 adjusted features were filtered for heritability values above 0.4, which resulted in 573 highly heritable features for fruit shape and size. Feature selection for shape and size resulted in 16 selected features showing various distributions (Table 1, Supplementary Fig. 1a in Supplementary File 1). Among the selected features, one was derived from images of the top view of fruits, ten accounted for standard deviation within the individual fruit shape or size of the different side views and five represented the average of the fruit shape or size measures from the side views. A comparison of principal components (PCs) derived from the 573 highly heritable features with the 16 selected features and the supplementary traits measured by the sorting machine showed that the PC1 was positively correlated with the sorting machine traits fruit length, volume, and single fruit weight (Fig. 2a). The PC2 was strongly correlated with the symmetry features (e.g. Av_SSD_Pol_SD_R_Sym.rel) and the shape heterogeneity features defined as standard deviation between measures of different fruit parts derived from polar data of the fruit side views (e.g. Av_SSD_Pol_SD_B.LR_Max). For the highly heritable features, the PC1 and PC2 explained 48.2 and 11.9% of the variance, respectively (Supplementary Fig. 2 in Supplementary File 1). The PC3 was strongly positively correlated with the feature Av_Top_Pol_Av_A.LR_RatM, which is a parameter describing fruit roundness from top view and negatively correlated with the sorting machine traits related to fruit size (Fig. 2b). The PC4 was strongly correlated with a symmetry feature (Av_Sav_Pol_SD_B_Sym.rel) and the feature Av_SSD_Pol_Rat.Av_AB.LR_SD describing the symmetry and homogeneity of the fruit. The PC3 and PC4 explained 8.9 and 4.0% of the variance, respectively. The PC5 to PC7 explained less than 3% of the variance each (Supplementary Figs. 2 and 3 in Supplementary File 1).

### Skin color features

**Fig. 3.**
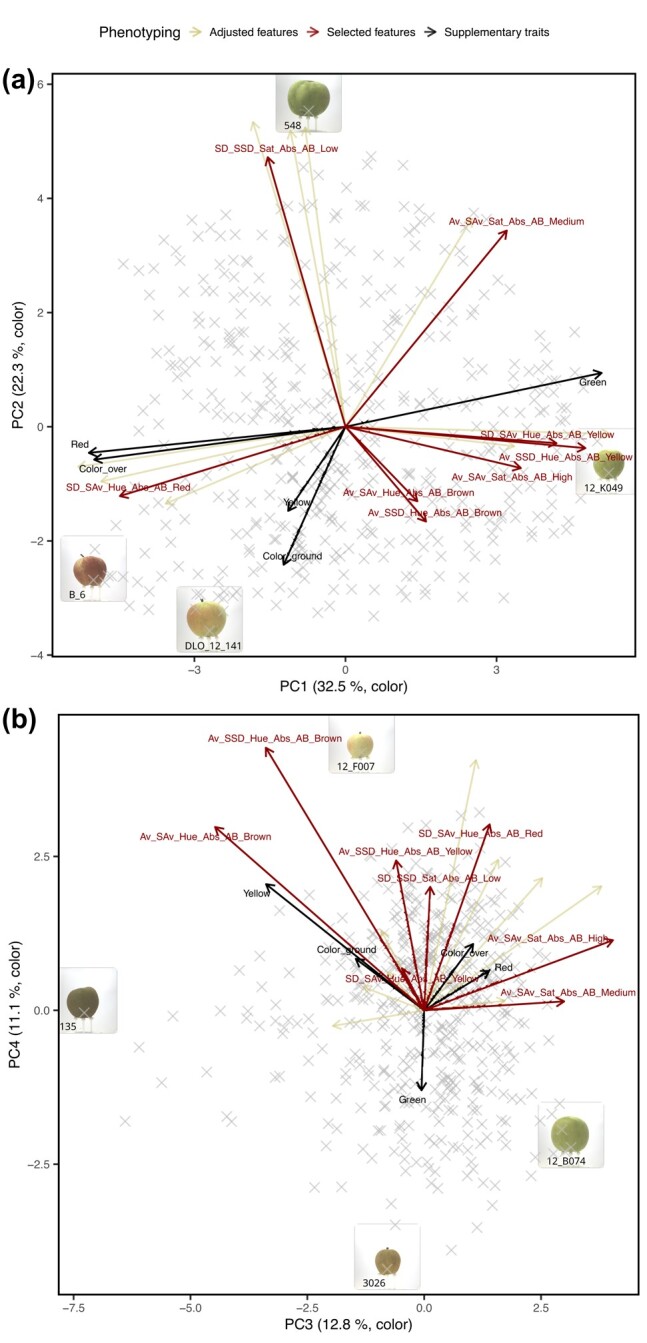
Principal component (PC) analysis of highly heritable features for color. The selected features for fruit color and supplementary features measured visually or using the sorting machine were overlayed in a biplot. Images of extreme genotypes of the apple REFPOP were shown in boxes and labeled with their respective genotype codes. a) PC1 and PC2, b) PC3 and PC4.

Filtering 20 adjusted features for heritability (*H*^2^*>* 0.4) resulted in 17 highly heritable fruit skin color features. From the highly heritable features, eight selected features were identified, and they represented all color feature groups (including the low, medium, and high saturation, as well as red, brown, and yellow hue features, Table 1). The selected features for color showed various distributions (Supplementary Fig. 1b in Supplementary File 1). In a PCA of the highly heritable features, the features were well represented by PC1 and PC2 that accounted for 54.8% of the variance in the data (Fig. 3a). When PCs derived from the highly heritable features were compared with the selected features and the supplementary traits measured by the sorting machine or scored visually, the biplot showed that PC1 was strongly positively correlated with the green color assessed by the sorting machine and the selected features for yellow hue (Fig. 3a). Furthermore, the PC1 was strongly negatively correlated with the red color scored visually and by the sorting machine as well as the selected feature representing red hue. The PC2 was strongly positively correlated with the selected feature for low saturation and negatively correlated with the visually scored ground color (discriminating between green and yellow ground color). The PC3 was strongly correlated with the color saturation selected features (i.e. Av_SAv_Sat_Abs_AB_Medium and Av_SAv_Sat_Abs_AB_High) (Fig. 3b). The PC4 was correlated with the sorting machine trait green and the selected features related to low saturation and yellow hue (e.g. Av_SSD_Hue_Abs_AB_Yellow). The selected features for brown hue (Av_SSD_Hue_Abs_AB_Brown and Av_SAv_Hue_Abs_AB_Brown) were well represented by the plane of PC3 and PC4 as shown by the long arrows, but these features did not strongly correlate with either of the PCs. The proportion of variance explained by PC3 and PC4 was 12.8 and 11.1%, respectively (Supplementary Fig. 2 in Supplementary File 1).

### GWAS for shape and size

**Fig. 4.**
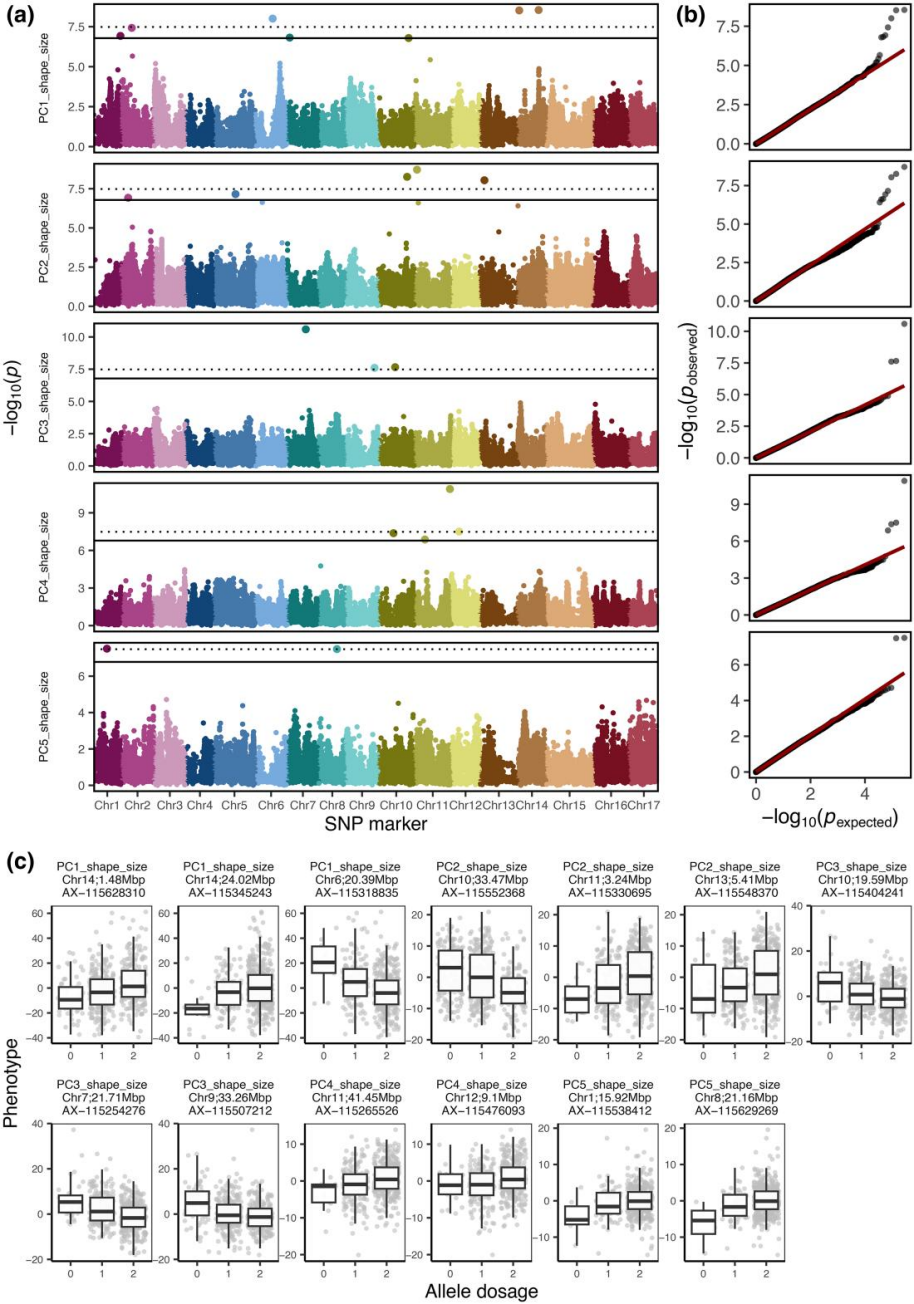
Results of the genome-wide association studies for the first five principal components (PCs) built from the highly heritable fruit shape and size features. a) Manhattan plots with the −log_10_(*P*) value for every SNP marker and PC. The SNP markers are displayed according to their physical position on the genome and chromosome (Chr). The Bonferroni-corrected significance level is indicated at 5 and 1% by the solid and dotted line, respectively. b) The expected and observed *P*-values for SNP markers and PCs. The 1:1 line for nonsignificant *P-*values is shown in red. c) The boxplots for allele dosage effect (0, 1, or 2 alternative alleles) for SNPs significantly associated with PCs at 1% significance level.

In total, 21 SNPs were significantly associated with PC1 to PC5 (Fig. 4a, Supplementary Fig. 4 in Supplementary File 1 and Supplementary Table 1 in Supplementary File 1). The plot of expected and observed −log_10_(*P*) values showed no apparent *P-*value inflation and therefore a good control of population structure (Fig. 4b). Out of the SNPs significantly associated with PC1 to PC5, 13 SNPs were observed at 1% significance level and showed mainly additive effects of the allele dosage (0, 1, or 2 alternative alleles) on the phenotype (Fig. 4c). Different proportions of phenotypic variance were explained by the SNPs associated with PCs (Table 2). Seven SNPs associated with PC1 explained together 35.7% of the phenotypic variance with additive effects for each SNP explaining between 2.8 and 9.2% of the variance. The five SNPs significantly associated with PC2 explained 21.1% of the variance with additive effects for each SNP explaining between 0.3 and 6.4% of the variance. The SNPs associated with PC3 to PC5 explained 18.5, 18.4, and 3.9% of the phenotypic variance, respectively. The PC6 and PC7 did not show any significant associations with SNP markers (Supplementary Fig. 5 in Supplementary File 1).

**Table 2.** Significant marker associations with principal component (PC) 1 to 5 for shape and size.

PC	Marker	Chr	Pos (bp)	*P*-value	Sum of squares	Explained variance (%)
PC1	AX-115460130	7	3,024,174	1.52E−07	3934.81	2.8
PC1	AX-115345243	14	24,020,772	2.87E−09	4295.028	3.1
PC1	AX-115227580	10	35,210,847	1.62E−07	5824.678	4.2
PC1	AX-115534322	2	12,130,252	3.75E−08	6050.312	4.3
PC1	AX-115628310	14	1,478,099	3.03E−09	8193.44	5.9
PC1	AX-115318835	6	20,393,412	9.86E−09	8516.722	6.1
PC1	AX-115380250	1	31,575,453	1.19E−07	12,866.27	9.2
PC1	Residual				89,512.3	64.3
PC2	AX-115490525	2	7,904,059	1.20E−07	112.5119	0.3
PC2	AX-115548370	13	5,411,038	9.11E−09	915.1389	2.8
PC2	AX-115429431	5	25,209,696	2.13E−08	1888.73	5.8
PC2	AX-115330695	11	3,241,944	1.93E−09	1921.867	5.9
PC2	AX-115552368	10	33,469,975	5.50E−09	2090.653	6.4
PC2	Residual				25,874.43	78.9
PC3	AX-115404241	10	19,586,733	2.20E−08	767.3217	3
PC3	AX-115507212	9	33,255,077	2.48E−08	948.2588	3.8
PC3	AX-115254276	7	21,705,520	2.58E−11	2957.387	11.7
PC3	Residual				20,563.65	81.5
PC4	AX-115659654	11	12,416,234	1.35E−07	434.6513	3.8
PC4	AX-115476093	12	9,098,303	3.16E−08	441.0631	3.9
PC4	AX-115419379	10	17,661,953	4.24E−08	591.8564	5.2
PC4	AX-115265526	11	41,448,132	1.26E−11	633.1262	5.6
PC4	Residual				9294.302	81.6
PC5	AX-115538412	1	15,918,343	3.09E−08	124.1923	1.5
PC5	AX-115629269	8	21,161,287	3.25E−08	197.7971	2.4
PC5	Residual				7864.159	96.1

Marker name, chromosome (Chr), position (Pos) in base pairs, *P*-value derived from GWAS, and the respective sum of squares and explained variance for each marker and the residual are shown.

Visual assessment of the effect of allele dosage on the side-view fruit contour for each associated SNP and PC showed major effects of individual SNPs on PCs (Supplementary Fig. 6 in Supplementary File 1 and Supplementary Table 1 in Supplementary File 1). SNPs significantly associated with PC1 affected fruit size but showed only a minor effect on fruit shape. The SNPs associated with PC2 and PC3 showed a major effect on fruit shape, with different allele dosage resulting in round or lengthened (conical) fruit shapes. Two SNPs associated with PC4 affected fruit size, one SNP resulted in contour of round or conical fruit shape and one SNP (AX−115419379) was associated with round or very long (cylindrical) fruit contour. The associations with PC5 influenced both the fruit size (AX−115629269) and conical shape (AX−115538412). For multilocus genotypes assembled from SNPs associated with each of the PCs, additive effects of multilocus genotypes on PCs were found, and visual differences in shape and/or size were observed for images of genotypes from the extremes of the PC distribution (Supplementary Figs. 7–10 in Supplementary File 1). The PC3 additionally showed patterns of heterogeneity in fruit shape from the top view (Supplementary Fig. 9 in Supplementary File 1).

For 15 out of 16 selected features for fruit shape and size, 48 associations of markers with features were identified, and they affected the conical shape, cylindrical shape, or size of the fruit (Supplementary Figs. 4 and 11 in Supplementary File 1 and Supplementary Table 1 in Supplementary File 1). All associations with the selected features referred to the side view of the cameras and contours. No significant associations with the selected feature for the top camera view were found, and therefore the visual inspection of the top-view contours was omitted. For multilocus genotypes assembled from SNPs associated with the selected feature for symmetry Av_SSD_Pol_SD_R_Sym.rel, additive effects of multilocus genotypes on the feature were found, and visual differences in fruit symmetry were observed for images of genotypes from the extremes of the feature distribution (Supplementary Fig. 12 in Supplementary File 1).

**Fig. 5.**
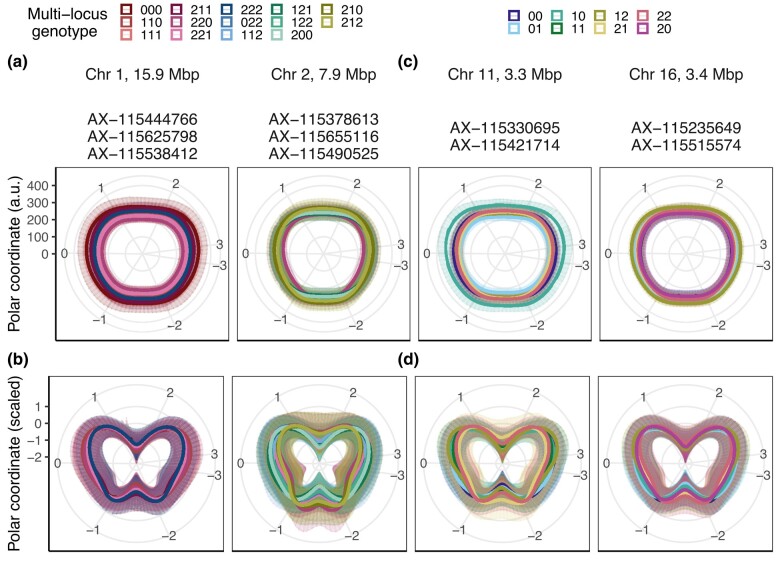
Effect of multilocus genotypes based on groups of physically linked SNPs for shape and size found in four genomic regions. The effect of each multilocus genotype on the averaged fruit contour is shown in a/c) absolute values and b/d) relative values. The relative values were scaled (mean = 0 and standard deviation = 1) for every individual apple fruit with the resulting minimum value being the center of the graph, i.e. the visualized diameter size is arbitrary, and the shape effect is enhanced. The error bars show the standard deviation of all genotypes included in the multilocus genotype (visible as semitransparent area around the fruit contour). The Bonferroni-corrected significance level of the considered associations was 5%. Multilocus genotypes were created separately for each chromosome (Chr) from the listed associated SNPs (SNP numbers prefixed with AX). To build the multilocus genotypes, allele dosages (0, 1, or 2 alternative alleles) of the associated SNPs were collapsed into combinations of allele dosages for each genotype. The SNPs were in the order of their positions on the reference genome used. The legend on the top left corresponds to subfigures a) and b), while the legend on the top right corresponds to subfigures c) and d).

Ten of the SNPs associated with PCs or selected features occurred physically close to each other (within 100,000 bp between pairs of SNPs) in four genomic regions with two or three SNPs located in each region. After these closely located SNPs were grouped into multilocus genotypes for each of the genomic regions (Fig. 5), the three SNPs on Chr 1 and 2 resulted in nine multilocus genotypes each, the two SNPs on Chr 11 and 16 in seven and eight multilocus genotypes, respectively. Although different multilocus genotypes of the four genomic regions showed an effect on both fruit shape and size, the differences in fruit size between multilocus genotypes of the genomic region on Chr 1 at 15.9 Mbp were particularly strong. In this region, the multilocus genotype “220” was associated with fruit contour of a notably small size. In the genomic region on Chr 2 at 7.9 Mbp, the multilocus genotypes “220”, “210”, and “212” resulted in decidedly conical shapes. The multilocus genotype “10” on Chr 11 at 3.3 Mbp showed an effect towards large fruit size. The multiocus genotypes on Chr 16 at 3.4 Mbp showed no pronounced effect on fruit shape or size.

### GWAS for skin color

**Fig. 6.**
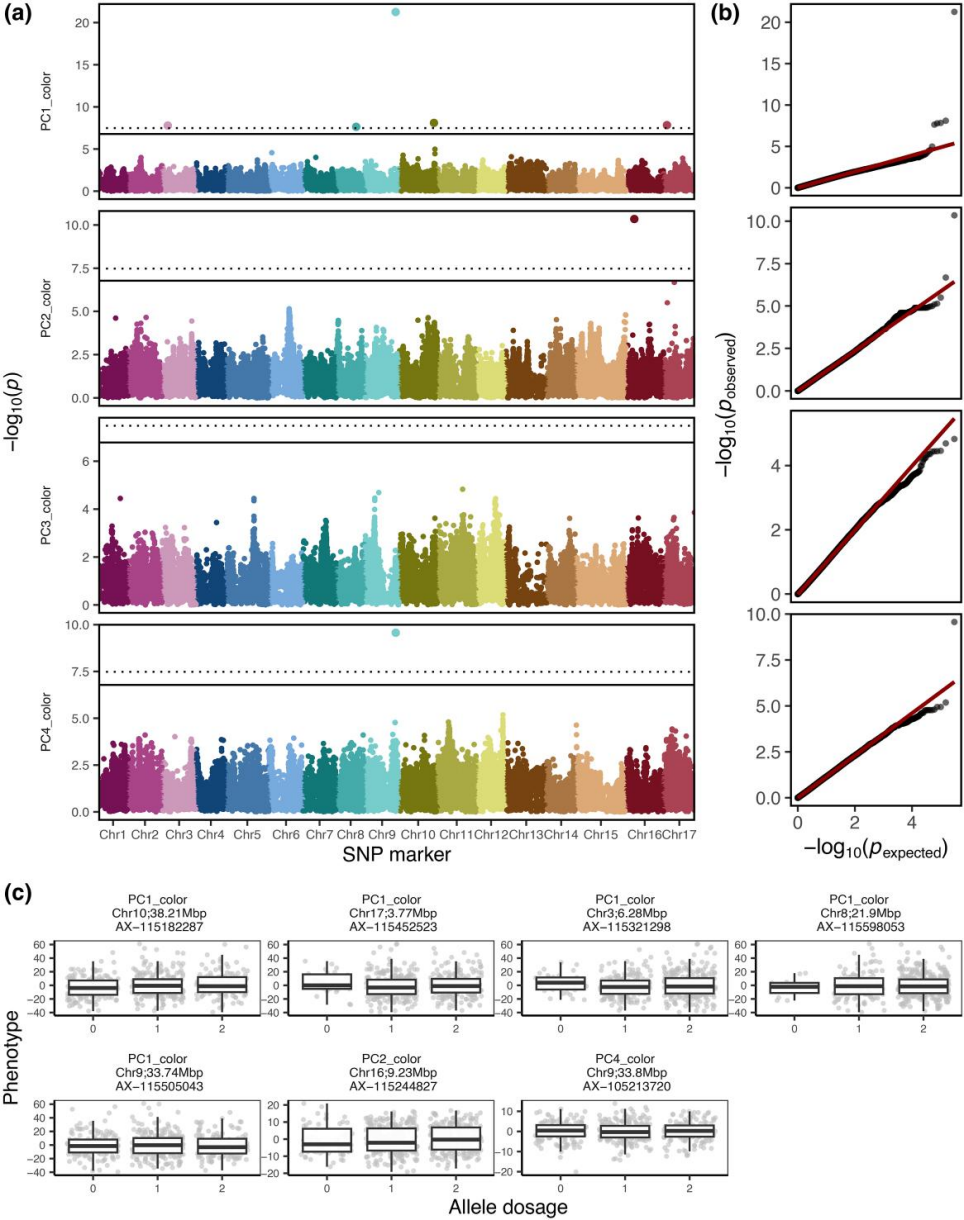
Results of the genome-wide association studies for the first four principal components (PCs) built from the highly heritable fruit color features. a) Manhattan plots with the −log_10_(*P*) value for every SNP marker and PC. The SNP markers are displayed according to their physical position on the genome and chromosome (Chr). The Bonferroni-corrected significance level is indicated at 5 and 1% by the solid and dotted line, respectively. b) The expected and observed *P-*values for SNP markers and PCs. The 1:1 line for nonsignificant *P-*values is shown in red. c) The boxplots for allele dosage effect (0, 1, or 2 alternative alleles) for SNPs significantly associated with PCs at 1% significance level.

**Fig. 7.**
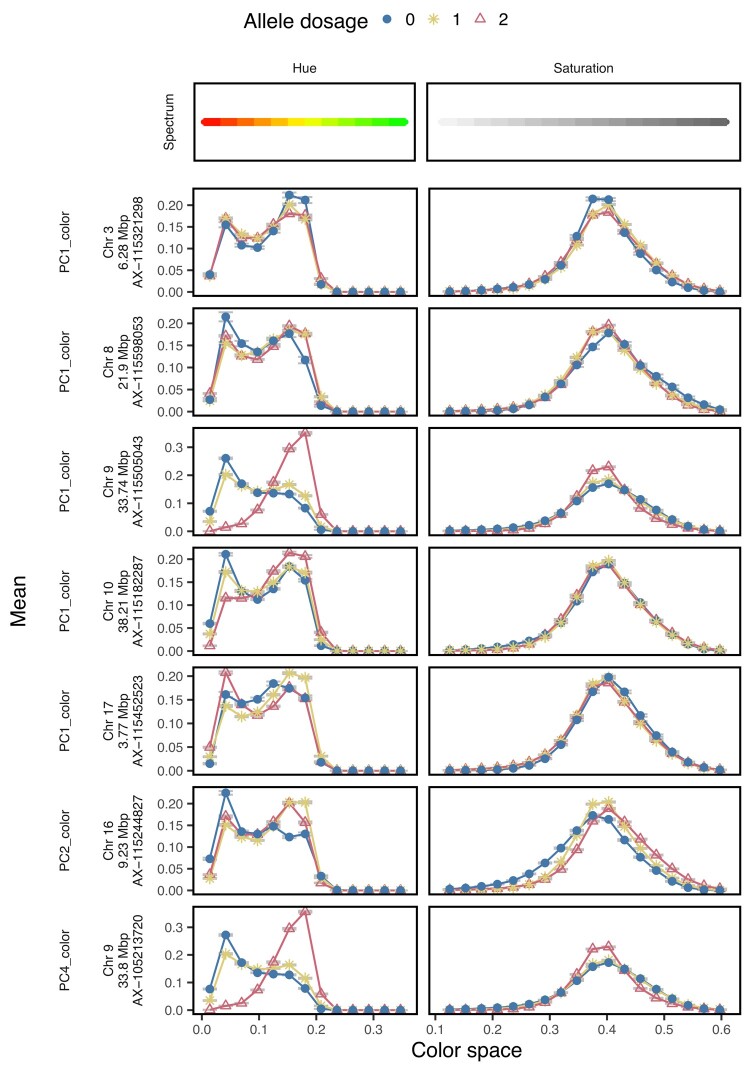
Allele dosage effect (0, 1, or 2 alternative alleles) of the SNPs significantly associated with the principal component (PC) 1, 2, and 4 on the hue and saturation color space. The error bars show the standard deviation of all genotypes per allele dosage. The Bonferroni-corrected significance level of the considered associations was 5%.

In total, 16 SNPs were significantly associated with three PCs and six out of the eight selected features for the apple fruit skin color (Supplementary Table 1 in Supplementary File 1). Seven SNPs were significantly associated with PC1, PC2 and PC4, while no significant association was observed for PC3 (Fig. 6a). No *P-*value inflation was visible in the Q–Q plot (Fig. 6b). All seven SNPs were identified above the 1% significance level and showed mainly additive effects on the color PCs (Fig. 6c). Upon visual inspection of major effects of allele dosage (0, 1, or 2 alternative alleles) that were assessed from feature distributions along the hue and saturation spectra (Fig. 7, Supplementary Fig. 13 in Supplementary File 1), alleles associated with red and light green hue or red and yellow hue were identified (Supplementary Table 1 in Supplementary File 1). Following this principle, five SNPs associated with PC1, PC4, medium saturation, high saturation, red and yellow hue in the genomic region on Chr 9 at 32.5–33.8 Mbp showed two peaks for red hue and one prominent peak for light green hue (Fig. 7, Supplementary Fig. 13 in Supplementary File 1). For saturation, all three allele dosages of the SNPs in the genomic region on Chr 9 showed distributions resembling bell curves of similar mean with an increased kurtosis for the allele associated with light green hue (Fig. 7, Supplementary Fig. 13 in Supplementary File 1). Additionally, similarities between distributions along the hue and saturation spectra were found for three SNPs associated with PC2 on Chr 16 at 9.23 Mbp and the selected feature for low saturation (SD_SSD_Sat_Abs_AB_Low) on Chr 2 at 20.51 Mbp and Chr 10 at 2.51 Mbp (Fig. 7, Supplementary Fig. 13 in Supplementary File 1). For these three SNPs, a peak was found in the yellow part of the hue spectrum and shifts of the distributions for the different allele dosages on the saturation spectrum towards low and high saturation were observed. The remaining seven SNPs associated with the color features showed only minor (undefinable) effects on the hue and saturation spectra (Fig. 7, Supplementary Fig. 13 in Supplementary File 1). For multilocus genotypes assembled from SNPs significantly associated with the selected feature for color SD_SAv_Hue_Abs_AB_Yellow, visual differences in fruit color were observed for images of genotypes from the extremes of the feature distribution (Supplementary Fig. 14 in Supplementary File 1).

### New and previously published associations

Out of 69 associations of markers with PCs and selected features for fruit shape and size and 16 associations of markers with PCs and selected features for fruit skin color that were identified in this study, 19 SNPs co-localized in genomic regions (genetic distance *<*0.1 Mbp) with 35 associations that have been reported in previous publications (Supplementary Table 2 in Supplementary File 1). Two markers (AX-105213720 and AX-115452523) identified in this study were identical as reported by Jung *et al*. (2022) and associated with green color and red overcolor. Additionally, 198 potential overlaps (corresponding to 48 SNP markers for fruit shape and size and 12 SNPs for color) were found between our and the previously reported loci (Supplementary Table 3 in Supplementary File 1). No overlapping positions with QTL reported in previous studies were found for 22 and three SNPs associated with shape/size and color features, respectively (Supplementary Table 4 in Supplementary File 1).

## Discussion

### Fruit shape and size

For over a decade, numerous studies have discovered QTL associated with apple fruit quality traits such as fruit shape and size (Kenis *et al*. 2008; Devoghalaere *et al*. 2012; Sun *et al*. 2012). The extensive collection of published fruit quality loci was recently reviewed by Jung *et al*. (2022). Making use of this review, we found several groups of three to four associated SNPs from our study that matched chromosome segments found in former studies and potentially pinpointed loci of broader interest (Supplementary Table 3 in Supplementary File 1). Such groups of SNPs associated with the selected features and PCs for fruit shape and size were located on Chr 9 at 31.2–33.3 Mbp, Chr 10 at 15.4–19.6 Mbp, and Chr 11 at 3.2–6.4 Mbp, and various QTL for fruit size have been previously reported in the proximity of these loci (Kenis *et al*. 2008; Sun *et al*. 2012; Chang *et al*. 2014; Liu *et al*. 2016; Liao *et al*. 2021; Minamikawa *et al*. 2021). Not only the traits characterizing fruit size but also fruit shape described as ratio between fruit length and diameter has been previously associated with QTL that co-localized with the associated genomic regions we identified on Chr 9 at 31.2–33.3 Mbp, Chr 10 at 15.4–19.6 Mbp, and Chr 10 at 33.5–35.2 Mbp (Sun *et al*. 2012; Chang *et al*. 2014). The numerous overlaps between the known QTL and the presently reported loci proved that FruitPhenoBox is a suitable tool for high-throughput phenotyping of fruit shape and size, and that the obtained features can be successfully linked with genomic information using GWAS.

For the apple REFPOP, Jung *et al*. (2022) found various across-location and location-specific associations with size-related traits, several of them located on Chr 11 (e.g. at 3.6 Mbp). Using a subset of the apple REFPOP genotypes grown in Spain, these results were recently extended with numerous associations of SNPs with traits describing fruit shape and size (Dujak and Aranzana 2023), and many of these associations were distributed along Chr 11. Dujak and Aranzana (2023) found a haploblock located at 4.5 Mbp on Chr 11, which contained strong associations with shape-related traits and two ovate family protein genes that were suggested as candidates for shape determination in apple. Despite the lack of exact overlap, the group of SNPs on Chr 11 at 3.2–6.4 Mbp and multilocus genotypes based on groups of physically linked SNPs at 3.3 Mbp reported in our study, which was performed for a subset of apple REFPOP genotypes grown in Switzerland, supported the importance of the genomic region on Chr 11 in regulation of fruit shape and size in apple. Validation of GWAS results using an independent population is necessary to verify the robustness of the identified genetic associations.

Out of all associations reported here for the selected features and PCs for fruit shape and size, 19 SNPs located across seven different chromosomes did not colocalize with QTL from the literature reviewed for fruit shape and size traits (Supplementary Table 4 in Supplementary File 1). Among the novel loci, a group of five SNPs on Chr 1 at 15.6–20.7 Mbp was associated with fruit size (four SNP markers) and conical shape (one SNP marker). Three of these SNPs occurred physically close to each other (within 100,000 bp), and the multilocus genotypes built with them showed a pronounced effect on fruit size (Fig. 5). The different multilocus genotypes provide with novel information about the genetic architecture of fruit size, and they could be used for marker-assisted selection for fruits of different sizes to explain a larger proportion of phenotypic variance than individual SNPs and to target selection at specific allelic combinations. To our knowledge and despite the numerous QTL known for size-related traits in apple (Kenis *et al*. 2008; Devoghalaere *et al*. 2012; Costa 2015; Duan *et al*. 2017), marker-assisted selection for fruit size has yet to be applied in apple. Markers selecting for fruit size may be especially helpful when breeding new varieties bearing resistances to various pests and diseases originating from small-fruit wild apple accessions (e.g. *M. floribunda* 821, the ornamental ‘Evereste’ or *Malus* × *robusta* 5). Selecting for multilocus genotypes associated with larger fruit size using marker-assisted selection could improve efficiency of breeding such resistant varieties.

For fruit shape, the novel associations with conical and cylindrical fruit shapes can assist in breeding for or against exotic fruit shapes in the future and offer a possibility to explore fruit shape beyond the traditionally studied fruit length/diameter ratio (Sun *et al*. 2012; Chang *et al*. 2014). Because of their association with round and prolonged shapes that were visible from the average fruit contours, the individual SNPs and multilocus genotypes built with SNPs of the newly discovered genomic region on Chr 2 at 7.6–7.9 Mbp can offer the opportunity for breeders to address round or conical fruit shapes (Fig. 5).

Despite the distinct effect of the newly discovered group of loci on Chr 2 at 7.6–7.9 Mbp on fruit shape, a minor effect of the multilocus genotypes on fruit size was observed from the averaged fruit contours (Fig. 5). Similar combination of effects on both fruit shape and size was visible for the novel group of loci on Chr 1 at 15.6–20.7 Mbp that was primarily associated with fruit size, as well as for the groups of loci on Chr 11 and 16 (Fig. 5). Loci associated simultaneously with shape and size have been shown in other crops such as cucurbits, tomatoes, or pear (Zhang *et al*. 2013; Pan *et al*. 2020; Sierra-Orozco *et al*. 2021). Although it was proposed earlier that size and shape are under independent genetic control in apple (Chang *et al*. 2014), the simultaneous effects of the loci described here on both fruit shape and size may suggest a biological interdependence between the shape and size of apple fruits.

### Fruit skin color

Many studies have explored the genetic mechanisms behind coloring of apple fruit, resulting in reports of QTL mostly located on Chr 9 (Bianco *et al*. 2014; Chagné *et al*. 2016; Duan *et al*. 2017; Moriya *et al*. 2017). For the associations with the color PCs and selected features found here (Supplementary Table 1 in Supplementary File 1), numerous overlaps in the bottom segment of Chr 9 at 32.5–33.8 Mbp were found between our associations and the associations found in the review of Jung *et al*. (2022) (Supplementary Table 3 in Supplementary File 1). These associations are all likely related to the degree of red overcolor, and they confirmed the role of the locus on Chr 9 in genetic regulation of red skin color in apples.

Although the locus on Chr 9 is known to explain the majority of the phenotypic variance in the red skin color of apples, additional minor loci on other chromosomes have been discovered for this trait (Duan *et al*. 2017; Jung *et al*. 2022). Our comparison with literature showed ten overlaps with associations for the selected features and PCs for color located on Chr 2 at 20.5 Mbp, Chr 6 at 3.1 Mbp, Chr 8 at 21.9 Mbp, Chr 10 at 2.5 Mbp, Chr 16 at 9.2 Mbp, and Chr 17 between 3.1 and 3.8 Mbp (Supplementary Table 3 in Supplementary File 1). The SNP on Chr 17 at 3.8 Mbp for PC1 co-localized exactly with a SNP reported for a visually screened red overcolor by Jung *et al*. (2022) (Supplementary Table 2 in Supplementary File 1). This supports the ability of the digital methods based on FruitPhenoBox to precisely localize QTL associated not only with shape and size features but also with color features. Furthermore, the FruitPhenoBox resulted in three associations with color features found on Chr 3 and 10 that did not colocalize with QTL from the reviewed literature (Supplementary Table 4 in Supplementary File 1) and therefore contributed novel insights into genetic architecture of color traits in apple.

Due to the peaks in the red part of the hue spectrum, which were observed for all rediscovered and novel associations with the color PCs and selected features (Fig. 7, Supplementary Fig. 13 in Supplementary File 1), it can be assumed that every reported SNP was associated with red skin color. At the same time, most of the SNPs were associated with similar patterns along the saturation spectrum. However, three SNPs associated with the selected feature for low saturation and PC2 located on Chr 2, 10, and 16 showed shifts in the distributions for the different allele dosages on the saturation spectrum towards higher or lower saturation (Fig. 7, Supplementary Fig. 13 in Supplementary File 1). Furthermore, PC2 was negatively correlated with ground color scored visually and yellow color scored by the sorting machine in Jung *et al*. (2022). PC2 was also positively correlated with the low saturation selected feature associated with the SNPs on Chr 2 and 10 (Fig. 3), and a peak was found in the yellow part of the hue spectrum for the SNPs on Chr 2, 10, and 16 (Fig. 7, Supplementary Fig. 13 in Supplementary File 1). Based on this evidence, the three SNPs may affect the variation in ground color, a trait that has been studied using visual assessment of the gradient between green and yellow skin color in the past (Costa 2015; Jung *et al*. 2022). The associations with the digitally estimated low saturation selected feature and PC2 contributed further understanding of the trait architecture towards the implementation of marker-based breeding technologies for ground color.

Strong correlations between PCs and the color- and size-related traits derived from the sorting machine suggest that PCs captured variation in these traits, highlighting the relationship between the PCs and the attributes assessed by the sorting machine. This finding underscores the utility of PCs in elucidating patterns and relationships within the dataset, particularly concerning traits relevant to the sorting machine’s measurements.

## Conclusion

The FruitPhenoBox provided accurate and detailed fruit phenotypes of a diverse reference population, which were used to discover associations of markers with principal components and selected features by GWAS. Digital phenotypes obtained with FruitPhenoBox showed as a powerful solution and efficient alternative to visual phenotyping while providing novel and rediscovered loci for fruit shape, size, and color, which enhanced our understanding of the variation and the genetics of the studied traits. Using marker-assisted selection, SNPs and multilocus genotypes associated with fruit size may improve the efficiency of breeding resistant varieties with resistances originating from small-fruited genotypes by reducing the number of pseudo-backcrosses needed to produce sufficiently large apples. Loci related to cylindrical or conical fruit shape can assist with breeding for or against exotic fruit shapes. Combined effects of discovered loci on both shape and size suggested genetic interdependence between these traits in apple. The minor loci associated with fruit color contributed novel understanding of trait architecture for the ground color of apple fruits, and they may complement the existing DNA tests for skin color of apples. The results of this study can support apple breeders when improving the process of selecting future apple varieties for their appearance by marker-assisted selection.

## Supplementary Material

jkae045_Supplementary_Data

## Data Availability

The images used in this study are available at https://doi.org/10.3929/ethz-b-000652840. The raw data extracted from the images and the derived features are available at https://doi.org/10.3929/ethz-b-000649762. All SNP genotypic data have been deposited at https://doi.org/10.15454/1ERHGX and https://doi.org/10.15454/IOPGYF. The raw supplementary phenotypic data are available at https://doi.org/10.15454/VARJYJ. The R code can be accessed through the following link https://gitlab.ethz.ch/kellebea/fruitphenobox. Supplemental material available at G3 online.
