# Supplementary material for: The genetic basis of apple shape and size unraveled by digital phenotyping
Source: G3 (Bethesda). 2024 Mar 5;14(5):jkae045. doi: 10.1093/g3journal/jkae045 (PMC11075547; doi:10.1093/g3journal/jkae045)
Supplement: jkae045_Supplementary_Data [file jkae045_supplementary_data.pdf]

Supplemental Materials

1 Supplemental tables

Table S1: Significant SNP associations with principal components (PCs) and selected features (r<0.75) for shape/size and color. Chromosome (Chr), position, p value, minor allele frequency (MAF), allele dosage, nonstandard (Nonstd) allele are shown. The explained variance is based on associated sum of squares regressing all identified SNPs of a trait (without correction for population structure).

Marker	Feature	Chr	Position	P value	MAF	Major effect	Allele dosage	Nonstd allele	Explained variance
AX-115444766	Av_SSD_Pol_Abs_B.L_SD	1	15580877	1.9×10^{-10}	0.05	size	0	T	0.08
AX-115444766	Av_SSD_X_Abs_A_Q25	1	15580877	7.4×10^{-11}	0.05	size	0	T	0.09
AX-115625798	Av_SSD_X_Abs_B_Q25	1	15868916	3.2×10^{-12}	0.06	size	0	C	0.10
AX-115538412	PC5_shape_size	1	15918343	3.1×10^{-8}	0.08	conical shape	2	T	0.01
AX-115221494	Av_SSD_X_Abs_A_Q25	1	17152361	9.6×10^{-10}	0.07	size	2	T	0.04
AX-115341839	Av_SAv_Pol_SD_A.LR_Min	1	20696409	1.5×10^{-7}	0.11	size	2	T	0.01
AX-115183496	Av_SSD_Pol_SD_B.LR_Q25	1	23578695	1.5×10^{-7}	0.08	cylindrical shape	0	C	0.02
AX-115380250	PC1_shape_size	1	31575453	1.2×10^{-7}	0.25	size	0	C	0.07
AX-115378613	Av_SAv_Pol_SD_A.LR_Min	2	7626325	1.5×10^{-7}	0.14	size	2	T	0.04
AX-115655116	Av_SSD_Pol_SD_B.LR_Q25	2	7872781	1.1×10^{-8}	0.15	size	2	A	0.00
AX-115490525	PC2_shape_size	2	7904059	1.2×10^{-7}	0.18	conical shape	0	A	0.00
AX-115534322	PC1_shape_size	2	12130252	3.7×10^{-8}	0.43	size	0	A	0.03
AX-115629265	Av_SSD_Pol_Rat.Av_AB.LR_Max	2	35251284	1.3×10^{-7}	0.06	conical shape	0	C	0.03
AX-115251897	Av_SSD_Pol_Abs_B.L_SD	3	2544800	1.3×10^{-8}	0.33	size	2	A	0.01
AX-115278645	Av_SSD_Pol_Rat.Av_AB.LR_Min	3	8512354	3.2×10^{-8}	0.26	size	2	T	0.04
AX-115451037	Av_SSD_Pol_Abs_AB_RatM	3	29264257	2.6×10^{-9}	0.49	undefinable			0.01
AX-115520624	Av_SSD_Pol_SD_B.LR_Q25	3	32236169	7.4×10^{-8}	0.37	size	2	A	0.04
AX-115506351	Av_SAv_Pol_SD_A.LR_Min	4	30194434	6×10^{-8}	0.07	size	1		0.07
AX-115459245	Av_SSD_Pol_Rat.Av_AB.LR_Max	5	5031575	7.6×10^{-9}	0.41	conical shape	0	G	0.07
AX-115429431	Av_SSD_Pol_SD_B.LR_Max	5	25209696	2.1×10^{-8}	0.41	conical shape	0	A	0.07
AX-115429431	PC2_shape_size	5	25209696	7×10^{-8}	0.41	conical shape	0	A	0.03
AX-115257677	Av_SAv_XY_Sum_A_MaxR	5	45121659	5.1×10^{-8}	0.39	conical shape	0	G	0.08
AX-115644112	Av_SSD_Pol_Rat.Av_AB.LR_SD	5	46356956	1.3×10^{-9}	0.11	conical shape	0	T	0.07
AX-115319264	Av_SSD_X_Abs_B_Q25	6	4761377	1.1×10^{-7}	0.05	size	0	A	0.04
AX-115375194	Av_SAv_XY_Sum_A_MaxR	6	8743643	6.4×10^{-14}	0.13	size	0	T	0.16
AX-115492426	Av_SSD_Pol_SD_B.LR_Max	6	10584742	9.9×10^{-9}	0.40	conical shape	2	T	0.04
AX-115318835	PC1_shape_size	6	20393412	9.9×10^{-9}	0.15	size	0	T	0.09
AX-115473970	Av_SSD_X_Abs_B_Q25	6	23085308	9×10^{-8}	0.14	size	0	A	0.06
AX-115460130	PC1_shape_size	7	3024174	1.5×10^{-7}	0.16	size	2	C	0.04
AX-115254491	Av_SAv_XY_Sum_A_MaxR	7	8648220	3.7×10^{-9}	0.35	size	1	G	0.01
AX-115254276	PC3_shape_size	7	21705520	2.6×10^{-11}	0.23	conical shape	2	T	0.12
AX-115457086	Av_SSD_Pol_Abs_AB_RatM	8	537872	1.8×10^{-8}	0.27	conical shape	0	G	0.04
AX-115445494	Av_SAv_Pol_Rat.Av_AB.LR_Q50	8	9562443	7.7×10^{-8}	0.07	conical shape	1	T	0.04
AX-115629269	PC5_shape_size	8	21161287	3.2×10^{-8}	0.06	size	0	A	0.02
AX-115442384	Av_SSD_X_Abs_A_Q25	9	4698417	1.2×10^{-8}	0.08	size	2	G	0.02
AX-115521252	Av_SSD_X_Abs_B_Q25	9	5170431	2.8×10^{-8}	0.45	size	1	G	0.04
AX-115391247	Av_SSD_Pol_SD_B.LR_Max	9	31184244	7.4×10^{-8}	0.33	conical shape	0	G	0.06
AX-115500353	Av_SSD_X_Abs_B_Q25	9	33000755	6.8×10^{-8}	0.29	conical shape	0	G	0.03
AX-115507212	PC3_shape_size	9	33255077	2.5×10^{-8}	0.35	conical shape	0	T	0.04
AX-115349165	Av_SSD_Pol_Rat.Av_AB.LR_Min	10	15436508	1.6×10^{-10}	0.11	cylindrical shape	0	T	0.06
AX-115419379	PC4_shape_size	10	17661953	4.2×10^{-8}	0.15	cylindrical shape	0	C	0.05
AX-115404241	PC3_shape_size	10	19586733	2.2×10^{-8}	0.25	conical shape	0	C	0.03
AX-115552368	PC2_shape_size	10	33469975	5.5×10^{-9}	0.47	conical shape	0	G	0.09
AX-115184639	Av_SSD_Pol_SD_B.LR_Q25	10	33907766	6.4×10^{-8}	0.37	conical shape	2	C	0.05
AX-115227580	PC1_shape_size	10	35210847	1.6×10^{-7}	0.30	size	0	G	0.04
AX-115330695	PC2_shape_size	11	3241944	1.9×10^{-9}	0.18	conical shape	2	G	0.05
AX-115421714	Av_SSD_Pol_SD_B.LR_Q25	11	3408716	1.1×10^{-8}	0.18	conical shape	2	A	0.04
AX-115327911	Av_SSD_Pol_Abs_AB_RatM	11	4706860	4.2×10^{-15}	0.39	size	0	G	0.07
AX-115449727	Av_SSD_Pol_SD_B.LR_Max	11	6367847	1.8×10^{-8}	0.25	conical shape	2	A	0.04
AX-115659654	PC4_shape_size	11	12416234	1.4×10^{-7}	0.19	conical shape	0	G	0.04
AX-115436422	Av_SSD_Pol_Abs_B.L_SD	11	15841357	9.3×10^{-10}	0.21	conical shape	0	G	0.03
AX-115482171	Av_SSD_Pol_Rat.Av_AB.LR_Min	11	33111593	1.8×10^{-9}	0.39	conical shape	0	A	0.05
AX-115440676	Av_SSD_Pol_Abs_B.L_SD	11	34816193	2.2×10^{-10}	0.05	size	0	C	0.03
AX-115300506	Av_SAv_Pol_Rat.Av_AB.LR_Q50	11	38069491	9.7×10^{-10}	0.17	conical shape	0	G	0.05
AX-115265526	PC4_shape_size	11	41448132	1.3×10^{-11}	0.19	conical shape	0	A	0.04
AX-115476093	PC4_shape_size	12	9098303	3.2×10^{-8}	0.20	size	0	T	0.06
AX-115548370	PC2_shape_size	13	5411038	9.1×10^{-9}	0.22	conical shape	2	G	0.03
AX-115389297	Av_SSD_Pol_SD_R_Sym.rel	13	13849729	9.6×10^{-10}	0.18	symmetric shape			0.06
AX-115287563	Av_SSD_Pol_Rat.Av_AB.LR_Max	13	14542354	6.2×10^{-8}	0.24	conical shape	1	T	0.05
AX-115628310	PC1_shape_size	14	1478099	3×10^{-9}	0.34	size	2	G	0.05
AX-115363036	Av_SSD_X_Abs_A_Q25	14	15862103	4.5×10^{-8}	0.17	size	0	T	0.10
AX-115311704	Av_SSD_Pol_Rat.Av_AB.LR_Min	14	21571993	7.6×10^{-10}	0.15	size	0	C	0.06
AX-115345243	PC1_shape_size	14	24020772	2.9×10^{-9}	0.14	size	2	C	0.03
AX-115461813	Av_SSD_Pol_SD_R_Sym.rel	15	10680808	3.3×10^{-10}	0.32	symmetric shape			0.07
AX-115631329	Av_SAv_Pol_Rat.Av_AB.LR_Q50	15	47545121	7.8×10^{-8}	0.09	conical shape	2	T	0.03
AX-115283778	Av_SSD_Pol_SD_B.LR_Max	15	48233842	2.4×10^{-8}	0.11	conical shape	0	C	0.06
AX-115235649	Av_SAv_Pol_Rat.Av_AB.LR_Q50	16	3230048	1.9×10^{-10}	0.39	size	2	A	0.06
AX-115515574	Av_SSD_Pol_SD_R_Sym.rel	16	3452384	2.3×10^{-8}	0.46	symmetric shape			0.06
AX-115375737	Av_SSD_Pol_Abs_B.L_SD	17	9806448	1.2×10^{-7}	0.48	size	2	A	0.06
AX-115316726	SD_SSD_Sat_Abs_AB_Low	2	20512335	1.5×10^{-9}	0.30	yellow	0	A	0.09
AX-115321298	PC1_color	3	6282194	1.6×10^{-8}	0.26	undefinable			0.05
AX-115210619	Av_SSD_Hue_Abs_AB_Yellow	6	3069025	1.2×10^{-8}	0.47	undefinable			0.11
AX-115598053	PC1_color	8	21899808	2.3×10^{-8}	0.14	undefinable			0.03
AX-115425415	Av_SAv_Sat_Abs_AB_Medium	9	32473327	3.4×10^{-8}	0.46	green	2	G	0.09
AX-115619261	Av_SAv_Sat_Abs_AB_High	9	32475582	1×10^{-9}	0.22	green	0	G	0.06
AX-115332892	SD_SAv_Hue_Abs_AB_Yellow	9	33729410	1.3×10^{-16}	0.49	green	2	C	0.15
AX-115505043	PC1_color	9	33744382	5.6×10^{-22}	0.46	green	2	G	0.33
AX-105213720	PC4_color	9	33801013	2.7×10^{-10}	0.48	green	2	C	0.10
AX-105213720	SD_SAv_Hue_Abs_AB_Red	9	33801013	9.9×10^{-35}	0.49	green	2	C	0.42
AX-115228698	SD_SSD_Sat_Abs_AB_Low	10	2507193	2.2×10^{-9}	0.41	yellow	2	C	0.05
AX-115182287	PC1_color	10	38213067	7.9×10^{-9}	0.47	undefinable			0.01
AX-115536962	SD_SSD_Sat_Abs_AB_Low	10	41138070	9.3×10^{-8}	0.34	undefinable			0.01
AX-115244827	PC2_color	16	9227750	4.5×10^{-11}	0.37	yellow	2	T	0.08
AX-115394842	SD_SAv_Hue_Abs_AB_Yellow	17	3024389	1.5×10^{-8}	0.25	undefinable			0.03
AX-115452523	PC1_color	17	3769755	1.5×10^{-8}	0.31	undefinable			0.02

Table S2: Overlap between QTL identified in the current and previous studies. The physical difference between the positions is given in bp. Only differences below 100’000 bp were considered.

Chr	Position	Marker	Position_ref	Marker_ref	Feature	Publication	Difference	Trait_ref	Remark
1	15868916	AX-115625798	15867063	Chr01_15867063	Av_SSD_X_Abs_B_Q25	Liao et al. (2021)	1853	Oxalate	2 years, China
1	15918343	AX-115538412	15906474	Chr01_15906474	PC5_shape_size	Liao et al. (2021)	11869	Oxalate	2 years, China
1	23578695	AX-115183496	23552731	AX-115322263	Av_SSD_Pol_SD_B.LR_Q25	Jung et al. (2022)	25964	Number of fruits	location-specific CHE
1	23578695	AX-115183496	23578926	SNP_FB_0431170	Av_SSD_Pol_SD_B.LR_Q25	Minamikawa et al. (2021)	-231	Firm	Across years, Japan
2	7626325	AX-115378613	7721047	MDP0000843352	Av_SAv_Pol_SD_A.LR_Min	Duan et al. (2017)	-94722	Fruit development	Peroxidase
2	7626325	AX-115378613	7697242	MDP0000293312	Av_SAv_Pol_SD_A.LR_Min	Duan et al. (2017)	-70917	Fruit development	Phosphatidylserine decarboxylase proenzyme 2
2	7872781	AX-115655116	7881043	MDP0000327940	Av_SSD_Pol_SD_B.LR_Q25	Duan et al. (2017)	-8262	Fruit development	Chlorophyll a-b binding protein, chloroplastic
2	7872781	AX-115655116	7869905	MDP0000232805	Av_SSD_Pol_SD_B.LR_Q25	Duan et al. (2017)	2876	Fruit development	Lipid transfer protein
2	7904059	AX-115490525	7883091	MDP0000232809	PC2_shape_size	Duan et al. (2017)	20968	Fruit development	3-hydroxyacyl-[acyl-carrier-protein] dehydratase FabZ
3	32236169	AX-115520624	32328637	AX-115621973	Av_SSD_Pol_SD_B.LR_Q25	Jung et al. (2022)	-92468	Floral emergence	location-specific BEL
4	30194434	AX-115506351	30124476	SNP_FB_0591255	Av_SAv_Pol_SD_A.LR_Min	Minamikawa et al. (2021)	69958	Mealiness	Across years, Japan
5	5031575	AX-115459245	4949273	AX-115392975	Av_SSD_Pol_Rat.Av_AB.LR_Max	Jung et al. (2022)	82302	Fruit firmness	location-specific CHE
5	25209696	AX-115429431	25236549	AX-115413471	Av_SSD_Pol_SD_B.LR_Max	Jung et al. (2022)	-26853	Flowering intensity	across-location
6	4761377	AX-115319264	4815618	AX-115220470	Av_SSD_X_Abs_B_Q25	Jung et al. (2022)	-54241	Russet freq. - overall	location-specific FRA
7	21705520	AX-115254276	21735820	MDP0000336563	PC3_shape_size	Duan et al. (2017)	-30300	Fruit development	Synovial sarcoma associated ss18 protein, putative
7	21705520	AX-115254276	21723408	MDP0000189381	PC3_shape_size	Duan et al. (2017)	-17888	Fruit development	Putative calmodulin-like protein
8	21161287	AX-115629269	21158378	MDP0000231982	PC5_shape_size	Duan et al. (2017)	2909	Fruit development	Pentatricopeptide repeat-containing protein
8	21161287	AX-115629269	21161135	MDP0000507227	PC5_shape_size	Duan et al. (2017)	152	Fruit development	UDP-glucose 4-epimerase, putative
10	15436508	AX-115349165	15373507	MDP0000161978	Av_SSD_Pol_Rat.Av_AB.LR_Min	Duan et al. (2017)	63001	Fruit development	Fatty acid desaturase, putative
16	3230048	AX-115235649	3220684	AX-115602744	Av_SAv_Pol_Rat.Av_AB.LR_Q50	Jung et al. (2022)	9364	Titratable acidity	location-specific CHE
16	3230048	AX-115235649	3229804	Chr16_3229804	Av_SAv_Pol_Rat.Av_AB.LR_Q50	Liao et al. (2021)	244	Malate-1	2 years, China
16	3452384	AX-115515574	3479443	SNP_FB_0336036	Av_SSD_Pol_SD_R_Sym.rel	Minamikawa et al. (2021)	-27059	CraTop	Across years, Japan
16	3452384	AX-115515574	3452384	Chr16_3452384	Av_SSD_Pol_SD_R_Sym.rel	Liao et al. (2021)	0	Malate-1	2 years, China
6	3069025	AX-115210619	3070483	AX-115210618	Av_SSD_Hue_Abs_AB_Yellow	Jung et al. (2022)	-1458	Red over color	location-specific ESP
9	33729410	AX-115332892	33801319	s9_33801319	SD_SAv_Hue_Abs_AB_Yellow	Duan et al. (2017)	-71909	Skin color	
9	33729410	AX-115332892	33799120	AX-115279459	SD_SAv_Hue_Abs_AB_Yellow	Jung et al. (2022)	-69710	Red over color	location-specific ITA
9	33801013	AX-105213720	33801013	AX-105213720	PC4_color	Jung et al. (2022)	0	Red over color	across-location
9	33801013	AX-105213720	33801013	AX-105213720	PC4_color	Jung et al. (2022)	0	Green color*	location-specific CHE
10	38213067	AX-115182287	38218534	AX-115263961	PC1_color	Jung et al. (2022)	-5467	Trunk diameter	location-specific FRA
10	38213067	AX-115182287	38217999	AX-115263958	PC1_color	Jung et al. (2022)	-4932	Green color*	location-specific CHE
10	41138070	AX-115536962	41129257	Chr10_41129257	SD_SSD_Sat_Abs_AB_Low	Liao et al. (2021)	8813	Citrate	2 years, China
16	9227750	AX-115244827	9244325	AX-115244807	PC2_color	Jung et al. (2022)	-16575	Ground color	across-location
16	9227750	AX-115244827	9240764	AX-115244813	PC2_color	Jung et al. (2022)	-13014	Harvest date	across-location
17	3024389	AX-115394842	3119571	AX-115231505	SD_SAv_Hue_Abs_AB_Yellow	Jung et al. (2022)	-95182	Water core grade*	location-specific ITA
17	3024389	AX-115394842	3096111	AX-115407463	SD_SAv_Hue_Abs_AB_Yellow	Jung et al. (2022)	-71722	Russet cover	location-specific ESP
17	3769755	AX-115452523	3823904	AX-115394824	PC1_color	Jung et al. (2022)	-54149	Red over color	location-specific BEL
17	3769755	AX-115452523	3769755	AX-115452523	PC1_color	Jung et al. (2022)	0	Red over color	location-specific ESP

Table S3: Identified markers with potentially overlapping positions reported in previous studies.

(continuous on next page)

Chr	Position	Segment	Marker	Feature	Publication	Trait in reference
1	23578695	bottom	AX-115183496	Av_SSD_Pol_SD_B.LR_Q25	Jung et al. (2022)	Number of fruits
1	23578695	bottom	AX-115183496	Av_SSD_Pol_SD_B.LR_Q25	Costa (2015)	Fruit height
1	31575453	bottom	AX-115380250	PC1_shape_size	Costa (2015)	Fruit height
2	35251284	bottom	AX-115629265	Av_SSD_Pol_Rat.Av_AB.LR_Max	Chang et al. (2014)	Fruit length, diameter or size
3	2544800	top	AX-115251897	Av_SSD_Pol_Abs_B.L_SD	Sun et al. (2015)	Fruit weight
3	8512354	top	AX-115278645	Av_SSD_Pol_Rat.Av_AB.LR_Min	Sun et al. (2015)	Fruit weight
3	29264257	bottom	AX-115451037	Av_SSD_Pol_Abs_AB_RatM	Chang et al. (2014)	Fruit length, diameter or size
3	29264257	bottom	AX-115451037	Av_SSD_Pol_Abs_AB_RatM	Potts et al. (2014)	Fruit diameter
3	29264257	bottom	AX-115451037	Av_SSD_Pol_Abs_AB_RatM	Potts et al. (2014)	Weight
3	29264257	bottom	AX-115451037	Av_SSD_Pol_Abs_AB_RatM	Potts et al. (2014)	Length
3	32236169	bottom	AX-115520624	Av_SSD_Pol_SD_B.LR_Q25	Chang et al. (2014)	Fruit length, diameter or size
3	32236169	bottom	AX-115520624	Av_SSD_Pol_SD_B.LR_Q25	Potts et al. (2014)	Fruit diameter
3	32236169	bottom	AX-115520624	Av_SSD_Pol_SD_B.LR_Q25	Potts et al. (2014)	Weight
3	32236169	bottom	AX-115520624	Av_SSD_Pol_SD_B.LR_Q25	Potts et al. (2014)	Length
5	5031575	top	AX-115459245	Av_SSD_Pol_Rat.Av_AB.LR_Max	Chang et al. (2014)	Ratio between height and diameter
5	5031575	top	AX-115459245	Av_SSD_Pol_Rat.Av_AB.LR_Max	Minamikawa et al. (2021)	Fruit weight in grams
5	25209696	center	AX-115429431	Av_SSD_Pol_SD_B.LR_Max	Potts et al. (2014)	Length
5	25209696	center	AX-115429431	PC2_shape_size	Potts et al. (2014)	Length
5	25209696	center	AX-115429431	Av_SSD_Pol_SD_B.LR_Max	Sun et al. (2015)	Fruit weight
5	25209696	center	AX-115429431	PC2_shape_size	Sun et al. (2015)	Fruit weight
5	25209696	center	AX-115429431	Av_SSD_Pol_SD_B.LR_Max	Potts et al. (2014)	Weight
5	25209696	center	AX-115429431	PC2_shape_size	Potts et al. (2014)	Weight
5	25209696	center	AX-115429431	Av_SSD_Pol_SD_B.LR_Max	Minamikawa et al. (2021)	Fruit weight in grams
5	25209696	center	AX-115429431	PC2_shape_size	Minamikawa et al. (2021)	Fruit weight in grams
5	25209696	center	AX-115429431	Av_SSD_Pol_SD_B.LR_Max	Potts et al. (2014)	Fruit diameter
5	25209696	center	AX-115429431	PC2_shape_size	Potts et al. (2014)	Fruit diameter
5	45121659	bottom	AX-115257677	Av_SAv_XY_Sum_A_MaxR	Chang et al. (2014)	Fruit length, diameter or size
5	46356956	bottom	AX-115644112	Av_SSD_Pol_Rat.Av_AB.LR_SD	Chang et al. (2014)	Fruit length, diameter or size
6	20393412	center	AX-115318835	PC1_shape_size	Liu et al. (2016)	Fruit weight
6	20393412	center	AX-115318835	PC1_shape_size	Liu et al. (2016)	Fruit diameter
6	23085308	center	AX-115473970	Av_SSD_X_Abs_B_Q25	Liu et al. (2016)	Fruit weight
6	23085308	center	AX-115473970	Av_SSD_X_Abs_B_Q25	Liu et al. (2016)	Fruit diameter
7	3024174	top	AX-115460130	PC1_shape_size	Costa (2015)	Fruit diameter
7	3024174	top	AX-115460130	PC1_shape_size	Costa (2015)	Fruit size (1-5 scale)
7	3024174	top	AX-115460130	PC1_shape_size	Costa (2015)	Weight
7	3024174	top	AX-115460130	PC1_shape_size	Costa (2015)	Fruit height
7	3024174	top	AX-115460130	PC1_shape_size	Chang et al. (2014)	Ratio between height and diameter
7	8648220	top	AX-115254491	Av_SAv_XY_Sum_A_MaxR	Costa (2015)	Fruit diameter
7	8648220	top	AX-115254491	Av_SAv_XY_Sum_A_MaxR	Costa (2015)	Fruit size (1-5 scale)
7	8648220	top	AX-115254491	Av_SAv_XY_Sum_A_MaxR	Costa (2015)	Weight
7	8648220	top	AX-115254491	Av_SAv_XY_Sum_A_MaxR	Costa (2015)	Fruit height
7	8648220	top	AX-115254491	Av_SAv_XY_Sum_A_MaxR	Chang et al. (2014)	Ratio between height and diameter
8	537872	top	AX-115457086	Av_SSD_Pol_Abs_AB_RatM	Chang et al. (2014)	Fruit length, diameter or size
8	9562443	top	AX-115445494	Av_SAv_Pol_Rat.Av_AB.LR_Q50	Chang et al. (2014)	Fruit length, diameter or size
8	21161287	bottom	AX-115629269	PC5_shape_size	Chang et al. (2014)	Fruit length, diameter or size
9	31184244	bottom	AX-115391247	Av_SSD_Pol_SD_B.LR_Max	Chang et al. (2014)	Ratio between height and diameter
9	31184244	bottom	AX-115391247	Av_SSD_Pol_SD_B.LR_Max	Chang et al. (2014)	Fruit length, diameter or size
9	33000755	bottom	AX-115500353	Av_SSD_X_Abs_B_Q25	Chang et al. (2014)	Ratio between height and diameter
9	33000755	bottom	AX-115500353	Av_SSD_X_Abs_B_Q25	Chang et al. (2014)	Fruit length, diameter or size
9	33255077	bottom	AX-115507212	PC3_shape_size	Chang et al. (2014)	Ratio between height and diameter
9	33255077	bottom	AX-115507212	PC3_shape_size	Chang et al. (2014)	Fruit length, diameter or size
10	15436508	center	AX-115349165	Av_SSD_Pol_Rat.Av_AB.LR_Min	Kenis et al. (2008)	Mean fruit fresh weight
10	15436508	center	AX-115349165	Av_SSD_Pol_Rat.Av_AB.LR_Min	Sun et al. (2012)	Ratio between height and diameter
10	15436508	center	AX-115349165	Av_SSD_Pol_Rat.Av_AB.LR_Min	Kenis et al. (2008)	Mean fruit diameter
10	17661953	center	AX-115419379	PC4_shape_size	Kenis et al. (2008)	Mean fruit fresh weight
10	17661953	center	AX-115419379	PC4_shape_size	Sun et al. (2012)	Ratio between height and diameter
10	17661953	center	AX-115419379	PC4_shape_size	Kenis et al. (2008)	Mean fruit diameter
10	19586733	center	AX-115404241	PC3_shape_size	Kenis et al. (2008)	Mean fruit fresh weight
10	19586733	center	AX-115404241	PC3_shape_size	Sun et al. (2012)	Ratio between height and diameter
10	19586733	center	AX-115404241	PC3_shape_size	Kenis et al. (2008)	Mean fruit diameter
10	33469975	bottom	AX-115552368	PC2_shape_size	Sun et al. (2012)	Ratio between height and diameter
10	33907766	bottom	AX-115184639	Av_SSD_Pol_SD_B.LR_Q25	Sun et al. (2012)	Ratio between height and diameter
10	35210847	bottom	AX-115227580	PC1_shape_size	Sun et al. (2012)	Ratio between height and diameter
11	3241944	top	AX-115330695	PC2_shape_size	Costa (2015)	Fruit size (1-5 scale)
11	3241944	top	AX-115330695	PC2_shape_size	Chang et al. (2014)	Ratio between height and diameter
11	3241944	top	AX-115330695	PC2_shape_size	Chang et al. (2014)	Fruit length, diameter or size
11	3241944	top	AX-115330695	PC2_shape_size	Sun et al. (2012)	Ratio between height and diameter
11	3408716	top	AX-115421714	Av_SSD_Pol_SD_B.LR_Q25	Costa (2015)	Fruit size (1-5 scale)
11	3408716	top	AX-115421714	Av_SSD_Pol_SD_B.LR_Q25	Chang et al. (2014)	Ratio between height and diameter
11	3408716	top	AX-115421714	Av_SSD_Pol_SD_B.LR_Q25	Chang et al. (2014)	Fruit length, diameter or size
11	3408716	top	AX-115421714	Av_SSD_Pol_SD_B.LR_Q25	Sun et al. (2012)	Ratio between height and diameter
11	4706860	top	AX-115327911	Av_SSD_Pol_Abs_AB_RatM	Costa (2015)	Fruit size (1-5 scale)
11	4706860	top	AX-115327911	Av_SSD_Pol_Abs_AB_RatM	Chang et al. (2014)	Ratio between height and diameter
11	4706860	top	AX-115327911	Av_SSD_Pol_Abs_AB_RatM	Chang et al. (2014)	Fruit length, diameter or size
11	4706860	top	AX-115327911	Av_SSD_Pol_Abs_AB_RatM	Sun et al. (2012)	Ratio between height and diameter
11	6367847	top	AX-115449727	Av_SSD_Pol_SD_B.LR_Max	Costa (2015)	Fruit size (1-5 scale)
11	6367847	top	AX-115449727	Av_SSD_Pol_SD_B.LR_Max	Chang et al. (2014)	Ratio between height and diameter
11	6367847	top	AX-115449727	Av_SSD_Pol_SD_B.LR_Max	Chang et al. (2014)	Fruit length, diameter or size
11	6367847	top	AX-115449727	Av_SSD_Pol_SD_B.LR_Max	Sun et al. (2012)	Ratio between height and diameter
11	12416234	top	AX-115659654	PC4_shape_size	Costa (2015)	Fruit size (1-5 scale)
11	12416234	top	AX-115659654	PC4_shape_size	Chang et al. (2014)	Ratio between height and diameter
11	12416234	top	AX-115659654	PC4_shape_size	Chang et al. (2014)	Fruit length, diameter or size
11	12416234	top	AX-115659654	PC4_shape_size	Sun et al. (2012)	Ratio between height and diameter
11	15841357	center	AX-115436422	Av_SSD_Pol_Abs_B.L_SD	Minamikawa et al. (2021)	Fruit weight in grams
11	15841357	center	AX-115436422	Av_SSD_Pol_Abs_B.L_SD	Sun et al. (2012)	Ratio between height and diameter
11	33111593	bottom	AX-115482171	Av_SSD_Pol_Rat.Av_AB.LR_Min	Sun et al. (2012)	Ratio between height and diameter
11	34816193	bottom	AX-115440676	Av_SSD_Pol_Abs_B.L_SD	Sun et al. (2012)	Ratio between height and diameter
11	38069491	bottom	AX-115300506	Av_SAv_Pol_Rat.Av_AB.LR_Q50	Sun et al. (2012)	Ratio between height and diameter
11	41448132	bottom	AX-115265526	PC4_shape_size	Sun et al. (2012)	Ratio between height and diameter
12	9098303	top	AX-115476093	PC4_shape_size	Sun et al. (2012)	Ratio between height and diameter
13	5411038	top	AX-115548370	PC2_shape_size	Minamikawa et al. (2021)	Fruit weight in grams
13	5411038	top	AX-115548370	PC2_shape_size	Sun et al. (2012)	Ratio between height and diameter
13	13849729	top	AX-115389297	Av_SSD_Pol_SD_R_Sym.rel	Minamikawa et al. (2021)	Fruit weight in grams
13	13849729	top	AX-115389297	Av_SSD_Pol_SD_R_Sym.rel	Sun et al. (2012)	Ratio between height and diameter
13	14542354	top	AX-115287563	Av_SSD_Pol_Rat.Av_AB.LR_Max	Minamikawa et al. (2021)	Fruit weight in grams
13	14542354	top	AX-115287563	Av_SSD_Pol_Rat.Av_AB.LR_Max	Sun et al. (2012)	Ratio between height and diameter
14	24020772	bottom	AX-115345243	PC1_shape_size	Chang et al. (2014)	Fruit length, diameter or size
15	10680808	top	AX-115461813	Av_SSD_Pol_SD_R_Sym.rel	Minamikawa et al. (2021)	Fruit weight in grams
15	10680808	top	AX-115461813	Av_SSD_Pol_SD_R_Sym.rel	Chang et al. (2014)	Ratio between height and diameter
15	10680808	top	AX-115461813	Av_SSD_Pol_SD_R_Sym.rel	Chang et al. (2014)	Fruit length, diameter or size
15	47545121	bottom	AX-115631329	Av_SAv_Pol_Rat.Av_AB.LR_Q50	Devoghalaere et al. (2012)	Fruit size - single fruit weight
15	48233842	bottom	AX-115283778	Av_SSD_Pol_SD_B.LR_Max	Devoghalaere et al. (2012)	Fruit size - single fruit weight
16	3230048	top	AX-115235649	Av_SAv_Pol_Rat.Av_AB.LR_Q50	Minamikawa et al. (2021)	Fruit weight in grams
16	3452384	top	AX-115515574	Av_SSD_Pol_SD_R_Sym.rel	Minamikawa et al. (2021)	Fruit weight in grams
17	9806448	top	AX-115375737	Av_SSD_Pol_Abs_B.L_SD	Costa (2015)	Fruit size (1-5 scale)
17	9806448	top	AX-115375737	Av_SSD_Pol_Abs_B.L_SD	Chang et al. (2014)	Ratio between height and diameter

Chr	Position	Segment	Marker	Feature	Publication	Trait in reference
17	9806448	top	AX-115375737	Av_SSD_Pol_Abs_B.L_SD	Costa (2015)	Fruit height
17	9806448	top	AX-115375737	Av_SSD_Pol_Abs_B.L_SD	Chang et al. (2014)	Fruit length, diameter or size
2	20512335	center	AX-115316726	SD_SSD_Sat_Abs_AB_Low	Duan et al. (2017)	Six levels of fruit skin color
6	3069025	top	AX-115210619	Av_SSD_Hue_Abs_AB_Yellow	Jung et al. (2022)	Red over color
8	21899808	bottom	AX-115598053	PC1_color	Minamikawa et al. (2021)	Degree of skin coloration in percent (1-5 scale)
9	32473327	bottom	AX-115425415	Av_SAv_Sat_Abs_AB_Medium	Costa (2015)	Overcolor %
9	32473327	bottom	AX-115425415	Av_SAv_Sat_Abs_AB_Medium	Amyotte et al. (2017)	Presence or absence of red skin colour
9	32473327	bottom	AX-115425415	Av_SAv_Sat_Abs_AB_Medium	Kunihisa et al. (2014)	“Depth” of the red coloration
9	32473327	bottom	AX-115425415	Av_SAv_Sat_Abs_AB_Medium	Migicovsky et al. (2021)	Red versus green
9	32473327	bottom	AX-115425415	Av_SAv_Sat_Abs_AB_Medium	Duan et al. (2017)	Six levels of fruit skin color
9	32473327	bottom	AX-115425415	Av_SAv_Sat_Abs_AB_Medium	Chagné et al. (2016)	Percentage overcolor
9	32473327	bottom	AX-115425415	Av_SAv_Sat_Abs_AB_Medium	Migicovsky et al. (2016)	Overcolor and overcolor intesity
9	32473327	bottom	AX-115425415	Av_SAv_Sat_Abs_AB_Medium	Lee et al. (2017)	Skin chroma (color intensity)
9	32473327	bottom	AX-115425415	Av_SAv_Sat_Abs_AB_Medium	Kumar et al. (2014)	Overcolor on zero to nine scale
9	32473327	bottom	AX-115425415	Av_SAv_Sat_Abs_AB_Medium	Minamikawa et al. (2021)	Degree of skin coloration in percent (1-5 scale)
9	32473327	bottom	AX-115425415	Av_SAv_Sat_Abs_AB_Medium	Moriya et al. (2017)	Overall color intensity
9	32473327	bottom	AX-115425415	Av_SAv_Sat_Abs_AB_Medium	McClure et al. (2018)	% red blush covering the surface
9	32473327	bottom	AX-115425415	Av_SAv_Sat_Abs_AB_Medium	Zheng et al. (2020)	Degree of fruit color cover
9	32475582	bottom	AX-115619261	Av_SAv_Sat_Abs_AB_High	Costa (2015)	Overcolor %
9	32475582	bottom	AX-115619261	Av_SAv_Sat_Abs_AB_High	Amyotte et al. (2017)	Presence or absence of red skin colour
9	32475582	bottom	AX-115619261	Av_SAv_Sat_Abs_AB_High	Kunihisa et al. (2014)	“Depth” of the red coloration
9	32475582	bottom	AX-115619261	Av_SAv_Sat_Abs_AB_High	Migicovsky et al. (2021)	Red versus green
9	32475582	bottom	AX-115619261	Av_SAv_Sat_Abs_AB_High	Duan et al. (2017)	Six levels of fruit skin color
9	32475582	bottom	AX-115619261	Av_SAv_Sat_Abs_AB_High	Chagné et al. (2016)	Percentage overcolor
9	32475582	bottom	AX-115619261	Av_SAv_Sat_Abs_AB_High	Migicovsky et al. (2016)	Overcolor and overcolor intesity
9	32475582	bottom	AX-115619261	Av_SAv_Sat_Abs_AB_High	Lee et al. (2017)	Skin chroma (color intensity)
9	32475582	bottom	AX-115619261	Av_SAv_Sat_Abs_AB_High	Kumar et al. (2014)	Overcolor on zero to nine scale
9	32475582	bottom	AX-115619261	Av_SAv_Sat_Abs_AB_High	Minamikawa et al. (2021)	Degree of skin coloration in percent (1-5 scale)
9	32475582	bottom	AX-115619261	Av_SAv_Sat_Abs_AB_High	Moriya et al. (2017)	Overall color intensity
9	32475582	bottom	AX-115619261	Av_SAv_Sat_Abs_AB_High	McClure et al. (2018)	% red blush covering the surface
9	32475582	bottom	AX-115619261	Av_SAv_Sat_Abs_AB_High	Zheng et al. (2020)	Degree of fruit color cover
9	33729410	bottom	AX-115332892	SD_SAv_Hue_Abs_AB_Yellow	Costa (2015)	Overcolor %
9	33729410	bottom	AX-115332892	SD_SAv_Hue_Abs_AB_Yellow	Amyotte et al. (2017)	Presence or absence of red skin colour
9	33729410	bottom	AX-115332892	SD_SAv_Hue_Abs_AB_Yellow	Kunihisa et al. (2014)	“Depth” of the red coloration
9	33729410	bottom	AX-115332892	SD_SAv_Hue_Abs_AB_Yellow	Migicovsky et al. (2021)	Red versus green
9	33729410	bottom	AX-115332892	SD_SAv_Hue_Abs_AB_Yellow	Duan et al. (2017)	Six levels of fruit skin color
9	33729410	bottom	AX-115332892	SD_SAv_Hue_Abs_AB_Yellow	Chagné et al. (2016)	Percentage overcolor
9	33729410	bottom	AX-115332892	SD_SAv_Hue_Abs_AB_Yellow	Migicovsky et al. (2016)	Overcolor and overcolor intesity
9	33729410	bottom	AX-115332892	SD_SAv_Hue_Abs_AB_Yellow	Lee et al. (2017)	Skin chroma (color intensity)
9	33729410	bottom	AX-115332892	SD_SAv_Hue_Abs_AB_Yellow	Kumar et al. (2014)	Overcolor on zero to nine scale
9	33729410	bottom	AX-115332892	SD_SAv_Hue_Abs_AB_Yellow	Minamikawa et al. (2021)	Degree of skin coloration in percent (1-5 scale)
9	33729410	bottom	AX-115332892	SD_SAv_Hue_Abs_AB_Yellow	Moriya et al. (2017)	Overall color intensity
9	33729410	bottom	AX-115332892	SD_SAv_Hue_Abs_AB_Yellow	McClure et al. (2018)	% red blush covering the surface
9	33729410	bottom	AX-115332892	SD_SAv_Hue_Abs_AB_Yellow	Zheng et al. (2020)	Degree of fruit color cover
9	33729410	bottom	AX-115332892	SD_SAv_Hue_Abs_AB_Yellow	Jung et al. (2022)	Red over color
9	33744382	bottom	AX-115505043	PC1_color	Amyotte et al. (2017)	Presence or absence of red skin colour
9	33744382	bottom	AX-115505043	PC1_color	Kunihisa et al. (2014)	“Depth” of the red coloration
9	33744382	bottom	AX-115505043	PC1_color	Kumar et al. (2014)	Overcolor on zero to nine scale
9	33744382	bottom	AX-115505043	PC1_color	Minamikawa et al. (2021)	Degree of skin coloration in percent (1-5 scale)
9	33744382	bottom	AX-115505043	PC1_color	Moriya et al. (2017)	Overall color intensity
9	33744382	bottom	AX-115505043	PC1_color	McClure et al. (2018)	% red blush covering the surface
9	33744382	bottom	AX-115505043	PC1_color	Zheng et al. (2020)	Degree of fruit color cover
9	33744382	bottom	AX-115505043	PC1_color	Costa (2015)	Overcolor %
9	33744382	bottom	AX-115505043	PC1_color	Jung et al. (2022)	Red over color
9	33744382	bottom	AX-115505043	PC1_color	Chagné et al. (2016)	Percentage overcolor
9	33744382	bottom	AX-115505043	PC1_color	Migicovsky et al. (2016)	Overcolor and overcolor intesity
9	33744382	bottom	AX-115505043	PC1_color	Migicovsky et al. (2021)	Red versus green
9	33744382	bottom	AX-115505043	PC1_color	Duan et al. (2017)	Six levels of fruit skin color
9	33744382	bottom	AX-115505043	PC1_color	Lee et al. (2017)	Skin chroma (color intensity)
9	33801013	bottom	AX-105213720	PC4_color	Jung et al. (2022)	Red over color
9	33801013	bottom	AX-105213720	SD_SAv_Hue_Abs_AB_Red	Jung et al. (2022)	Red over color
9	33801013	bottom	AX-105213720	PC4_color	Moriya et al. (2017)	Overall color intensity
9	33801013	bottom	AX-105213720	SD_SAv_Hue_Abs_AB_Red	Moriya et al. (2017)	Overall color intensity
9	33801013	bottom	AX-105213720	PC4_color	McClure et al. (2018)	% red blush covering the surface
9	33801013	bottom	AX-105213720	SD_SAv_Hue_Abs_AB_Red	McClure et al. (2018)	% red blush covering the surface
9	33801013	bottom	AX-105213720	PC4_color	Zheng et al. (2020)	Degree of fruit color cover
9	33801013	bottom	AX-105213720	SD_SAv_Hue_Abs_AB_Red	Zheng et al. (2020)	Degree of fruit color cover
9	33801013	bottom	AX-105213720	PC4_color	Costa (2015)	Overcolor %
9	33801013	bottom	AX-105213720	SD_SAv_Hue_Abs_AB_Red	Costa (2015)	Overcolor %
9	33801013	bottom	AX-105213720	PC4_color	Amyotte et al. (2017)	Presence or absence of red skin colour
9	33801013	bottom	AX-105213720	SD_SAv_Hue_Abs_AB_Red	Amyotte et al. (2017)	Presence or absence of red skin colour
9	33801013	bottom	AX-105213720	PC4_color	Kunihisa et al. (2014)	“Depth” of the red coloration
9	33801013	bottom	AX-105213720	SD_SAv_Hue_Abs_AB_Red	Kunihisa et al. (2014)	“Depth” of the red coloration
9	33801013	bottom	AX-105213720	PC4_color	Migicovsky et al. (2021)	Red versus green
9	33801013	bottom	AX-105213720	SD_SAv_Hue_Abs_AB_Red	Migicovsky et al. (2021)	Red versus green
9	33801013	bottom	AX-105213720	PC4_color	Duan et al. (2017)	Six levels of fruit skin color
9	33801013	bottom	AX-105213720	SD_SAv_Hue_Abs_AB_Red	Duan et al. (2017)	Six levels of fruit skin color
9	33801013	bottom	AX-105213720	PC4_color	Chagné et al. (2016)	Percentage overcolor
9	33801013	bottom	AX-105213720	SD_SAv_Hue_Abs_AB_Red	Chagné et al. (2016)	Percentage overcolor
9	33801013	bottom	AX-105213720	PC4_color	Migicovsky et al. (2016)	Overcolor and overcolor intesity
9	33801013	bottom	AX-105213720	SD_SAv_Hue_Abs_AB_Red	Migicovsky et al. (2016)	Overcolor and overcolor intesity
9	33801013	bottom	AX-105213720	PC4_color	Lee et al. (2017)	Skin chroma (color intensity)
9	33801013	bottom	AX-105213720	SD_SAv_Hue_Abs_AB_Red	Lee et al. (2017)	Skin chroma (color intensity)
9	33801013	bottom	AX-105213720	PC4_color	Kumar et al. (2014)	Overcolor on zero to nine scale
9	33801013	bottom	AX-105213720	SD_SAv_Hue_Abs_AB_Red	Kumar et al. (2014)	Overcolor on zero to nine scale
9	33801013	bottom	AX-105213720	PC4_color	Minamikawa et al. (2021)	Degree of skin coloration in percent (1-5 scale)
9	33801013	bottom	AX-105213720	SD_SAv_Hue_Abs_AB_Red	Minamikawa et al. (2021)	Degree of skin coloration in percent (1-5 scale)
10	2507193	top	AX-115228698	SD_SSD_Sat_Abs_AB_Low	Duan et al. (2017)	Six levels of fruit skin color
10	2507193	top	AX-115228698	SD_SSD_Sat_Abs_AB_Low	Zheng et al. (2020)	Degree of fruit color cover
16	9227750	top	AX-115244827	PC2_color	Jung et al. (2022)	Ground color
17	3024389	top	AX-115394842	SD_SAv_Hue_Abs_AB_Yellow	Jung et al. (2022)	Russet cover
17	3769755	top	AX-115452523	PC1_color	Jung et al. (2022)	Red over color

Table S4: Novel significant associations with principal components (PCs) and selected features (r<0.75) for shape/size and color identified in this study. These markers have no overlapping positions with QTL reported in previous studies.

Category	Chr	Position	Segment	Marker	Feature	Major effect
Size/shape	1	15580877	center	AX-115444766	Av_SSD_X_Abs_A_Q25	large size
Size/shape	1	15580877	center	AX-115444766	Av_SSD_Pol_Abs_B.L_SD	large size
Size/shape	1	15580877	center	AX-115444766	Av_SSD_X_Abs_A_Q25	large size
Size/shape	1	15580877	center	AX-115444766	Av_SSD_Pol_Abs_B.L_SD	large size
Size/shape	1	15868916	center	AX-115625798	Av_SSD_X_Abs_B_Q25	large size
Size/shape	1	15918343	center	AX-115538412	PC5_shape_size	conical shape
Size/shape	1	17152361	center	AX-115221494	Av_SSD_X_Abs_A_Q25	large size
Size/shape	1	20696409	center	AX-115341839	Av_SAv_Pol_SD_A.LR_Min	large size
Size/shape	2	7626325	top	AX-115378613	Av_SAv_Pol_SD_A.LR_Min	large size
Size/shape	2	7872781	top	AX-115655116	Av_SSD_Pol_SD_B.LR_Q25	large size
Size/shape	2	7904059	top	AX-115490525	PC2_shape_size	conical shape
Size/shape	2	12130252	top	AX-115534322	PC1_shape_size	large size
Size/shape	4	30194434	bottom	AX-115506351	Av_SAv_Pol_SD_A.LR_Min	large size
Size/shape	6	4761377	top	AX-115319264	Av_SSD_X_Abs_B_Q25	large size
Size/shape	6	8743643	top	AX-115375194	Av_SAv_XY_Sum_A_MaxR	large size
Size/shape	6	10584742	top	AX-115492426	Av_SSD_Pol_SD_B.LR_Max	conical shape
Size/shape	7	21705520	center	AX-115254276	PC3_shape_size	conical shape
Size/shape	9	4698417	top	AX-115442384	Av_SSD_X_Abs_A_Q25	large size
Size/shape	9	5170431	top	AX-115521252	Av_SSD_X_Abs_B_Q25	large size
Size/shape	14	1478099	top	AX-115628310	PC1_shape_size	large size
Size/shape	14	15862103	center	AX-115363036	Av_SSD_X_Abs_A_Q25	large size
Size/shape	14	21571993	center	AX-115311704	Av_SSD_Pol_Rat.Av_AB.LR_Min	large size
Color	3	6282194	top	AX-115321298	PC1_color	
Color	10	38213067	bottom	AX-115182287	PC1_color	
Color	10	41138070	bottom	AX-115536962	SD_SSD_Sat_Abs_AB_Low	

2 Supplemental figures

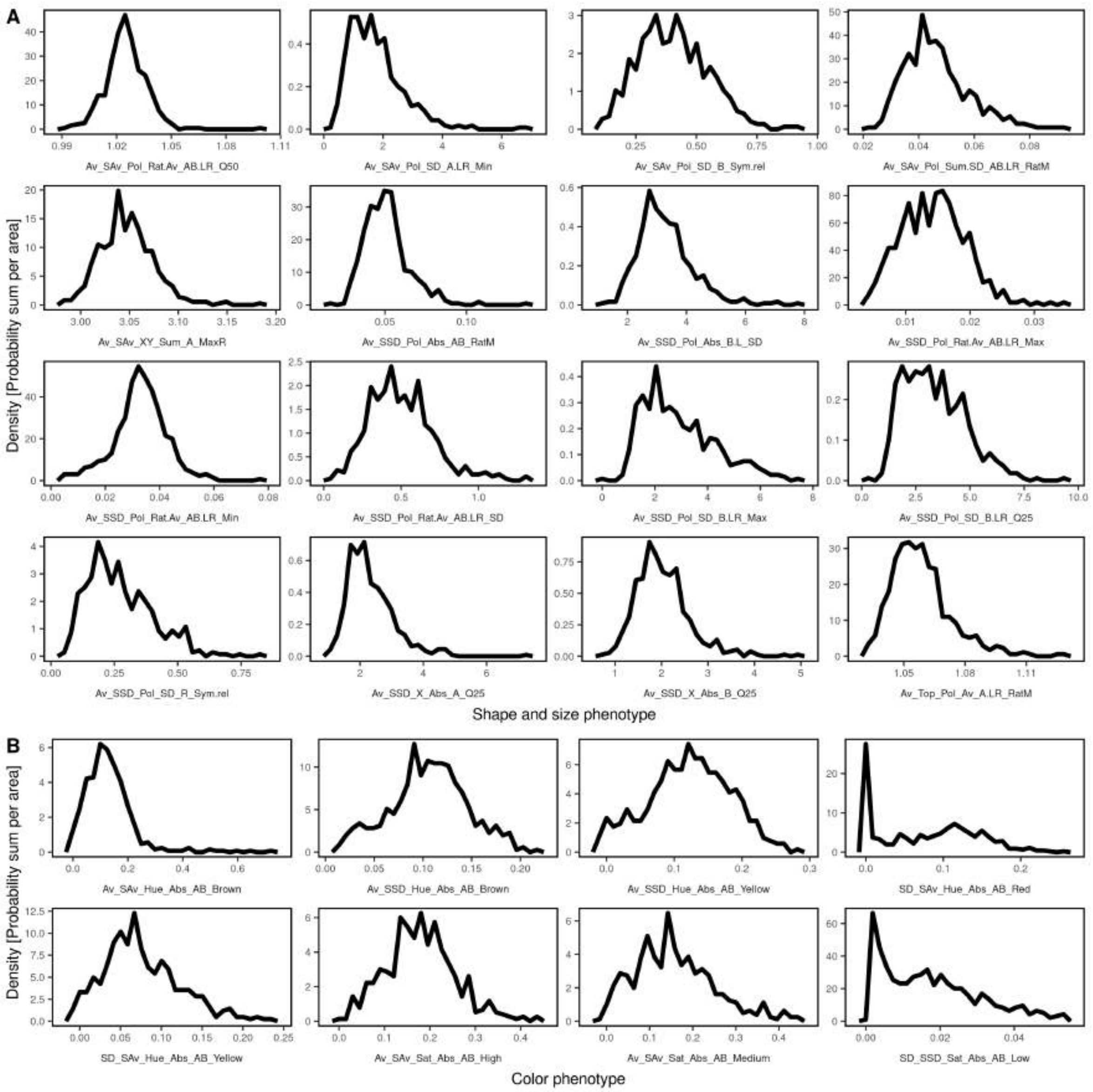

Figure S1: Kernel density estimate of the selected fruit features (r<0.75). A: Features for shape and size. B: Features for color. The estimate is a smoothed histogram with the integral equal to one calculated using a Gaussian kernel and a binwidth of 1/30 of the data range. See Table 1 for feature abbreviations and description.

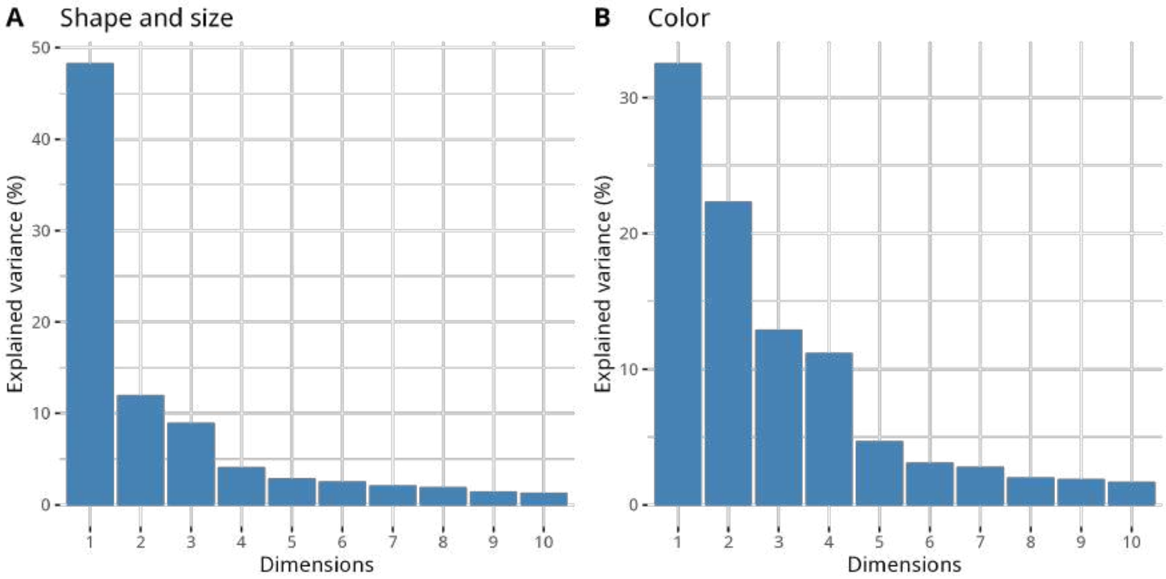

Figure S2: Proportions of variance explained by the first ten principal components derived from the highly heritable features for A: apple fruit shape and size, and B: skin color.

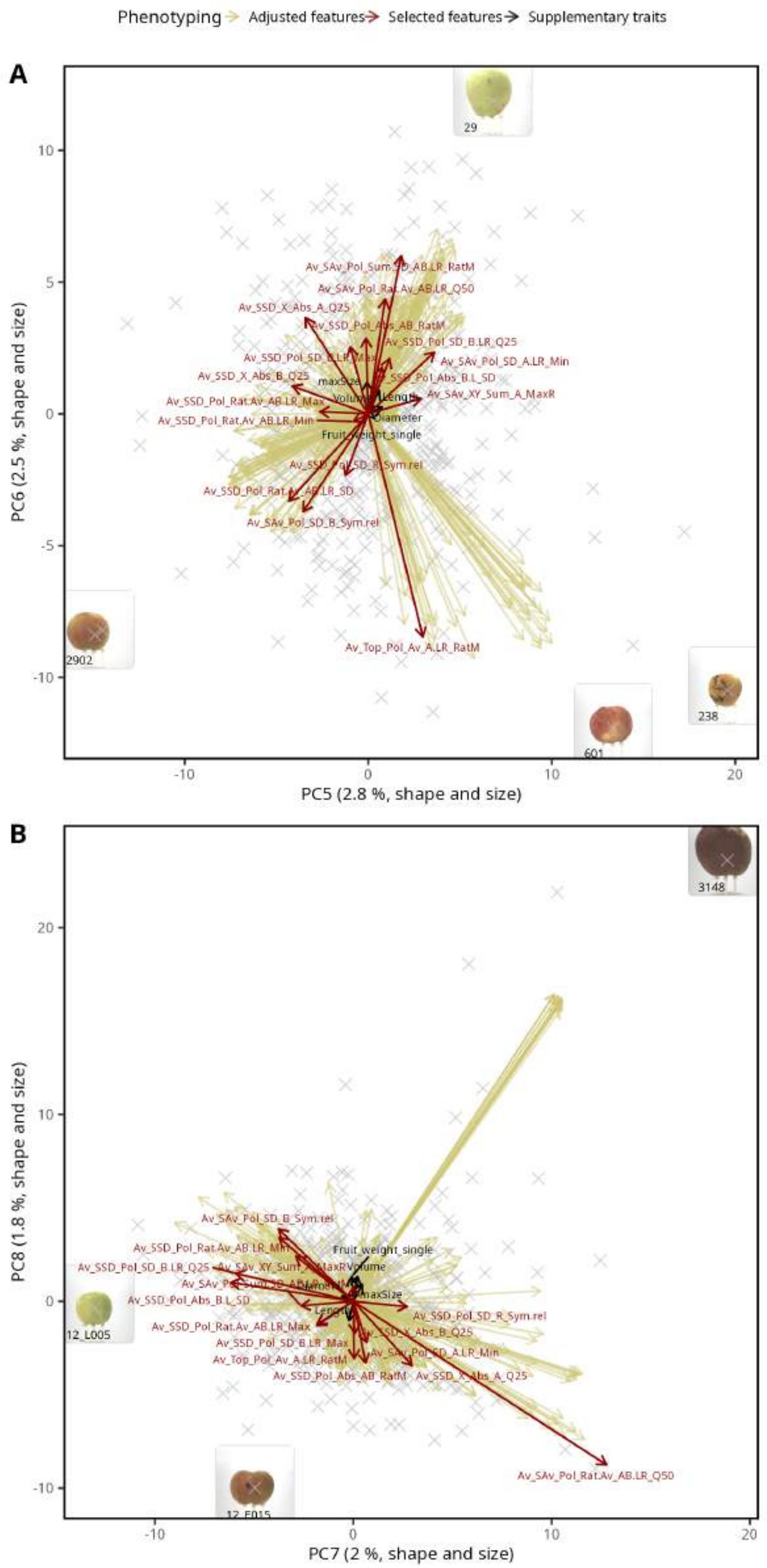

Figure S3: Principal component (PC) analysis of highly heritable features for fruit shape and size. Selected features (r<0.75) for fruit shape and size features (red arrows) and supplementary features measured using the sorting machine (black arrows) were overlayed in a biplot. Images of extreme genotypes of the apple REFPOP were shown in boxes and labeled with their respective genotype codes. A: PC5 and PC6, B: PC7 and PC8.

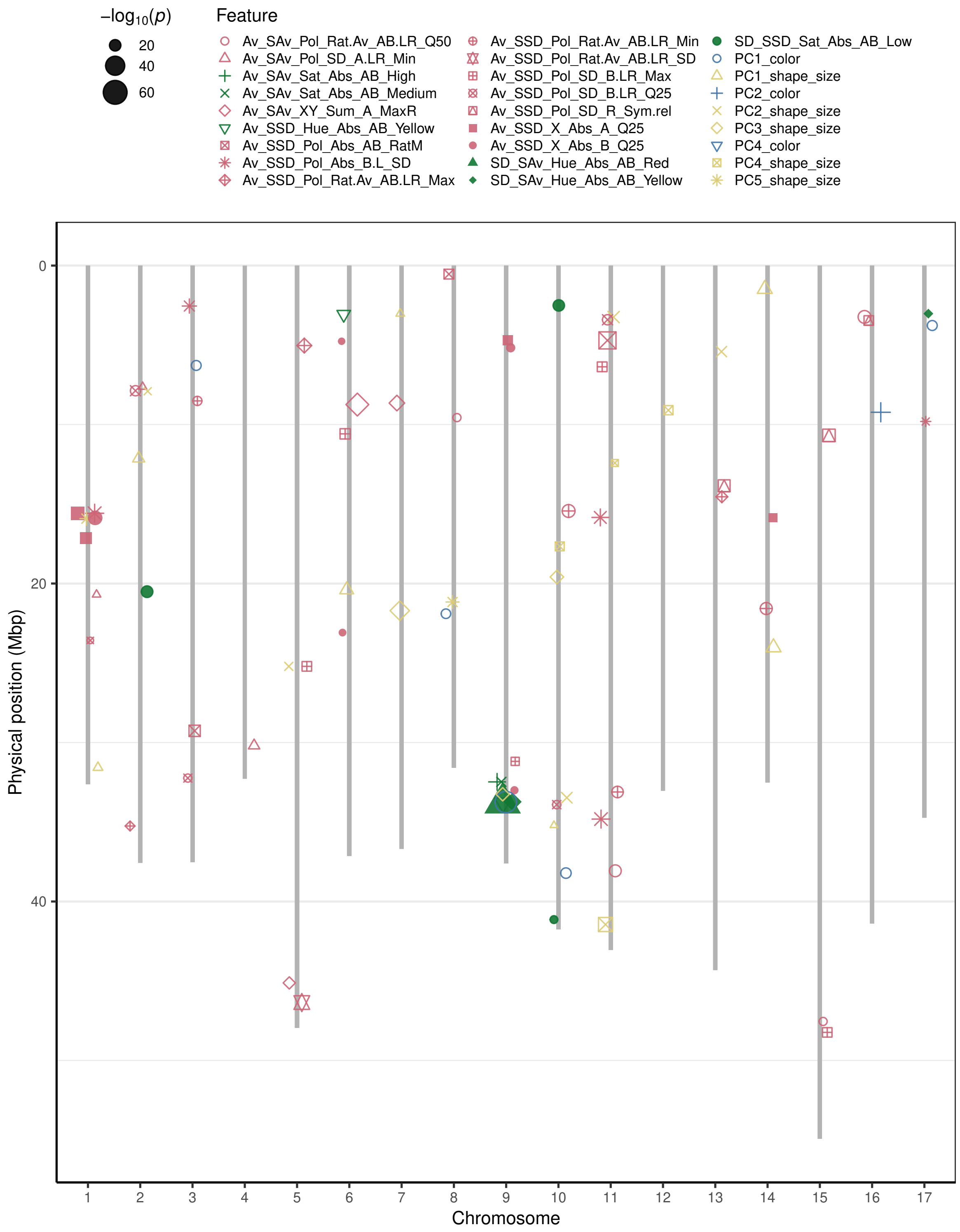

Figure S4: Distribution of the significant marker-trait associations on the apple genome. Associated SNPs were identified for the Phenobox color and size or shape features based on principal component analysis (PCA) or selected features (r<0.75). The size of the symbol represents the $-log_{10}(p)$ value.

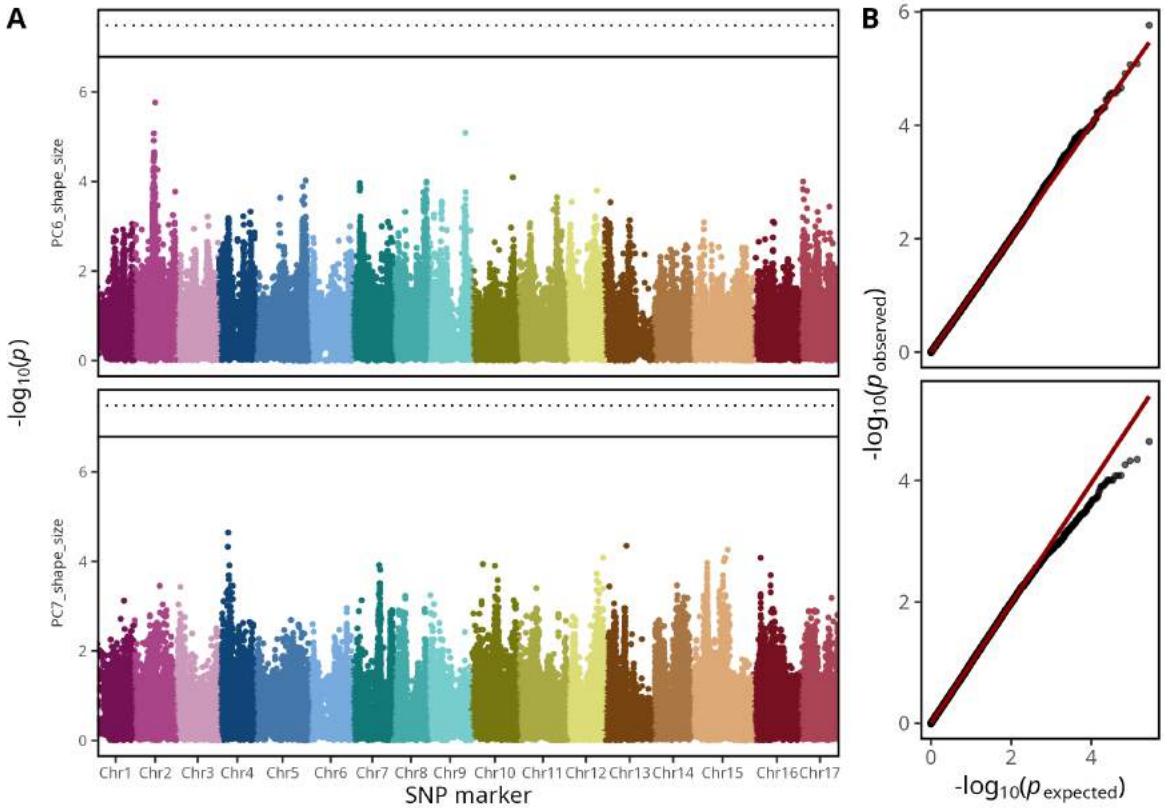

Figure S5: Results of the genome-wide association studies for the principal component (PC) 6 to 7 build from the highly heritable fruit shape and size features. A: Manhattan plots with the $-log_{10}(p)$ value for every SNP marker and PC. The SNP markers are displayed according to their physical position on the genome and chromosome (Chr). The Bonferroni-corrected significance level is indicated at 5% and 1% by the solid and dotted line, respectively. B: The expected and observed p-values for SNP markers and PCs. The 1:1 line for non-significant p-values is shown in red.

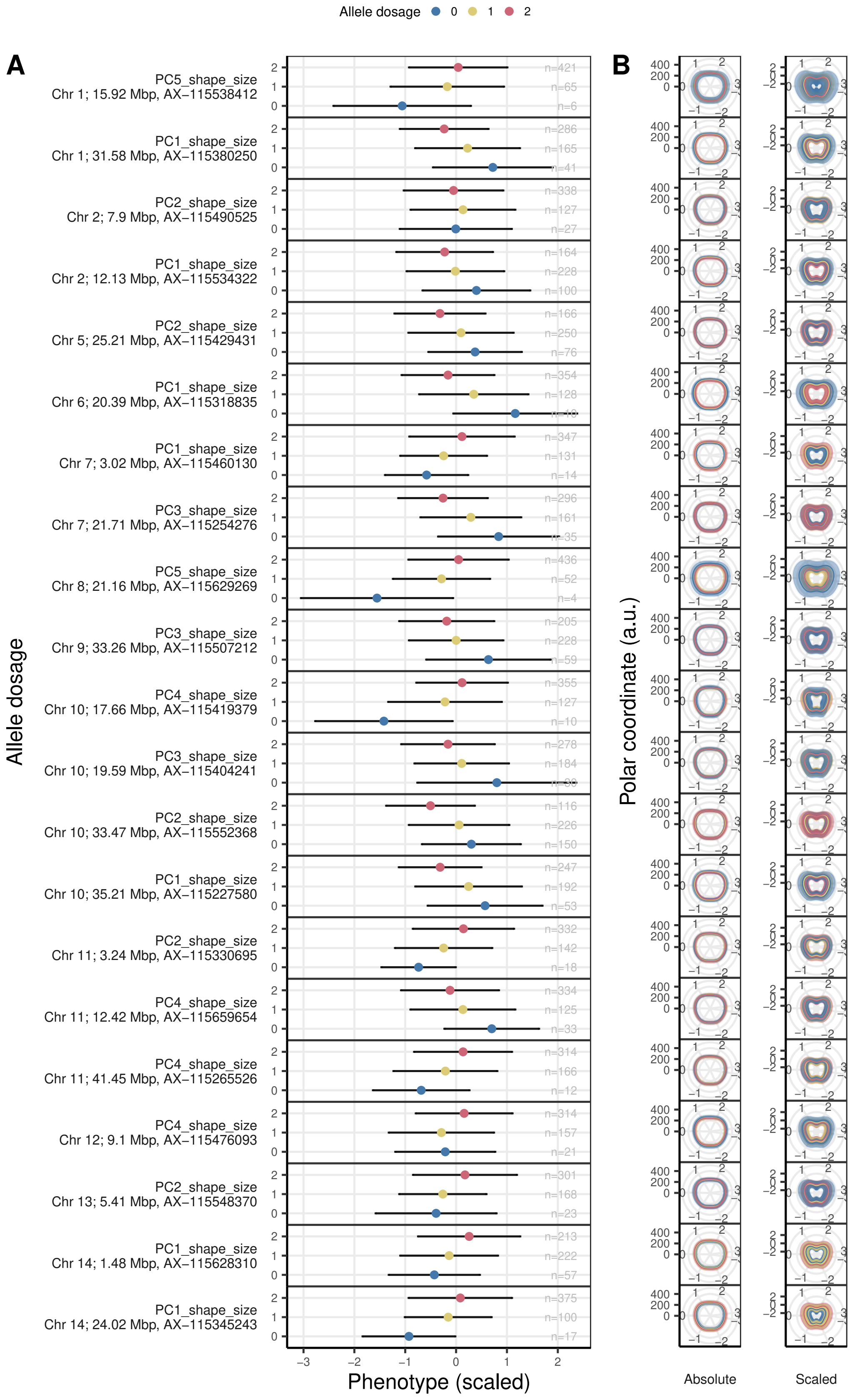

Figure S6: Effect of SNPs significantly associated with principal component (PC) 1 to 5 for fruit shape and size. A: Effect of the allele dosage (0, 1 or 2 alternative alleles) on the scaled values of PCs. B: The effect of each SNP on the averaged side-view fruit contour in absolute and relative values. The relative values were scaled (mean=0 and standard deviation=1) for every individual apple fruit with the resulting minimum value being the center of the graph, i.e., the visualized diameter size is arbitrary, and the shape effect is enhanced. The error bars show the standard deviation.

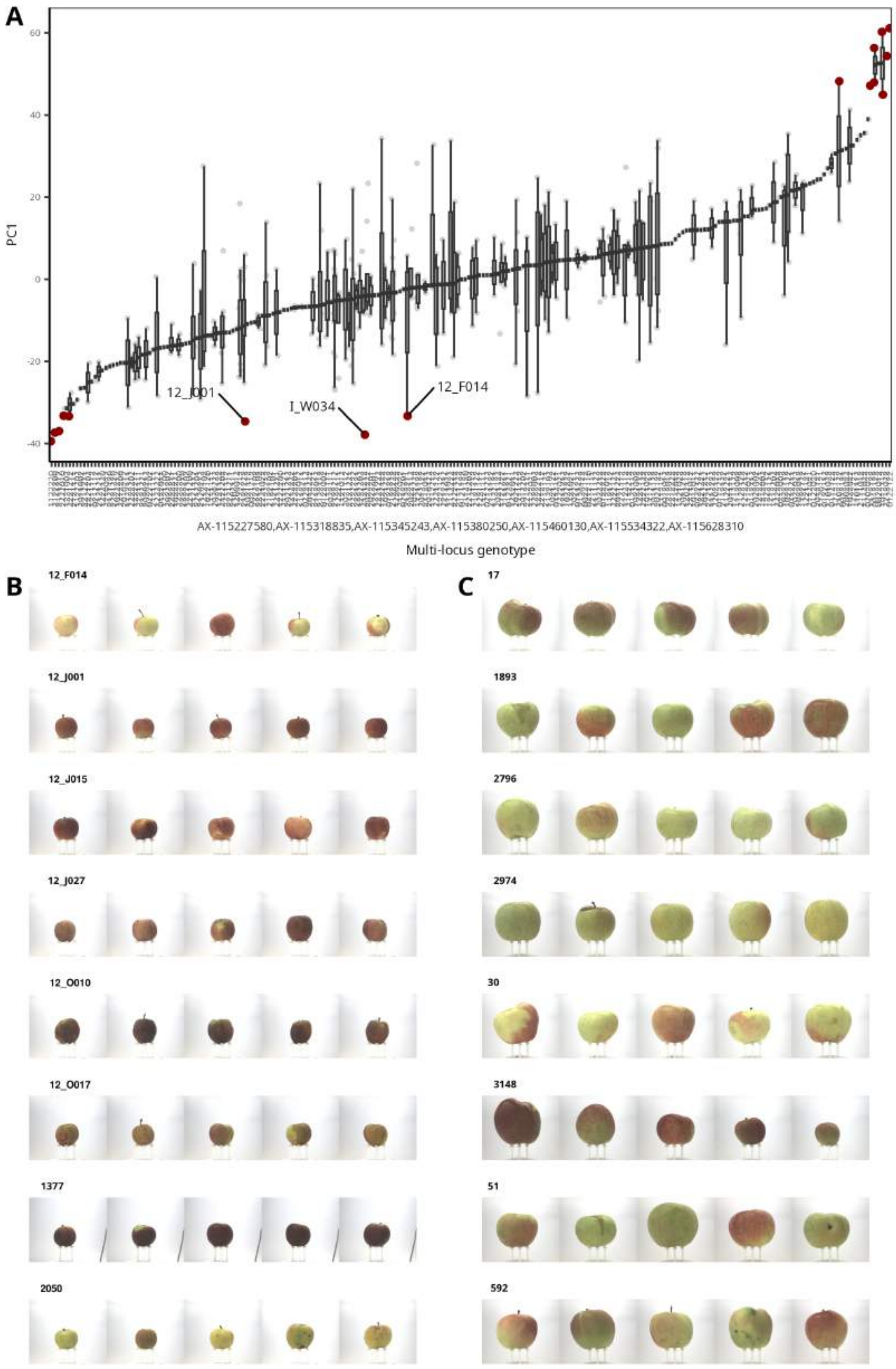

Figure S7: Combined effect of the SNPs identified for principal component 1 (PC1) on the size of apples. The SNPs were assembled into multi-locus genotypes. A: Boxplots of the effect of each multi-locus genotype on PC1. The multi-locus genotypes are labeled using combinations of allele dosage (0, 1 or 2 alternative alleles) of the SNPs. The eight most extreme phenotypes for low and high phenotypic trait values are depicted in red. Their fruit images with up to five replicates are shown in B: and C:, respectively.

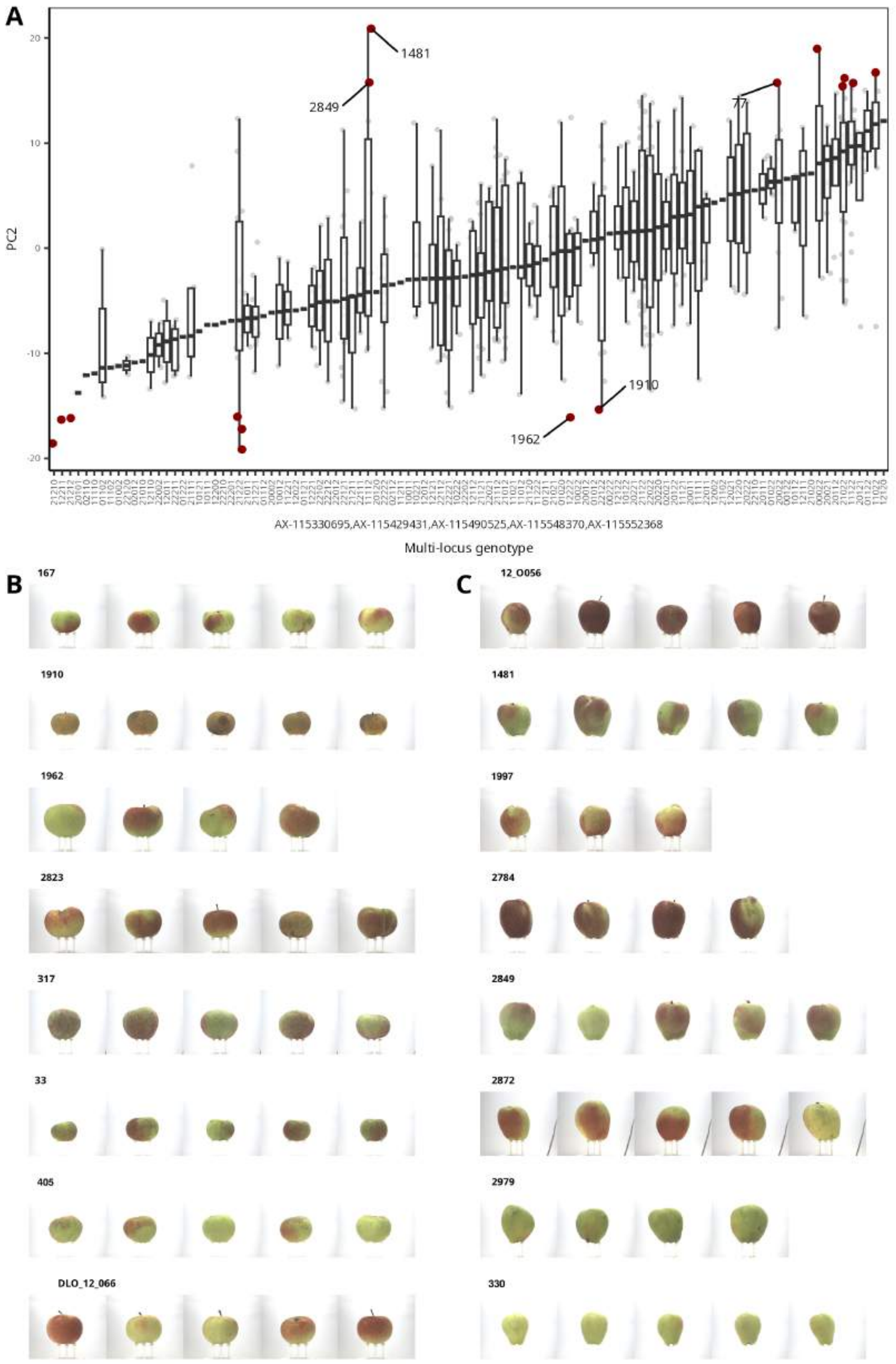

Figure S8: Combined effect of the SNPs identified for principal component 2 (PC2) on the shape of apples. The SNPs were assembled into multi-locus genotypes. A: Boxplots of the effect of each multi-locus genotype on PC2. The multi-locus genotypes are labeled using combinations of allele dosage (0, 1 or 2 alternative alleles) of the SNPs. The eight most extreme phenotypes for low and high phenotypic trait values are depicted in red. Their fruit images with up to five replicates are shown in B: and C:, respectively.

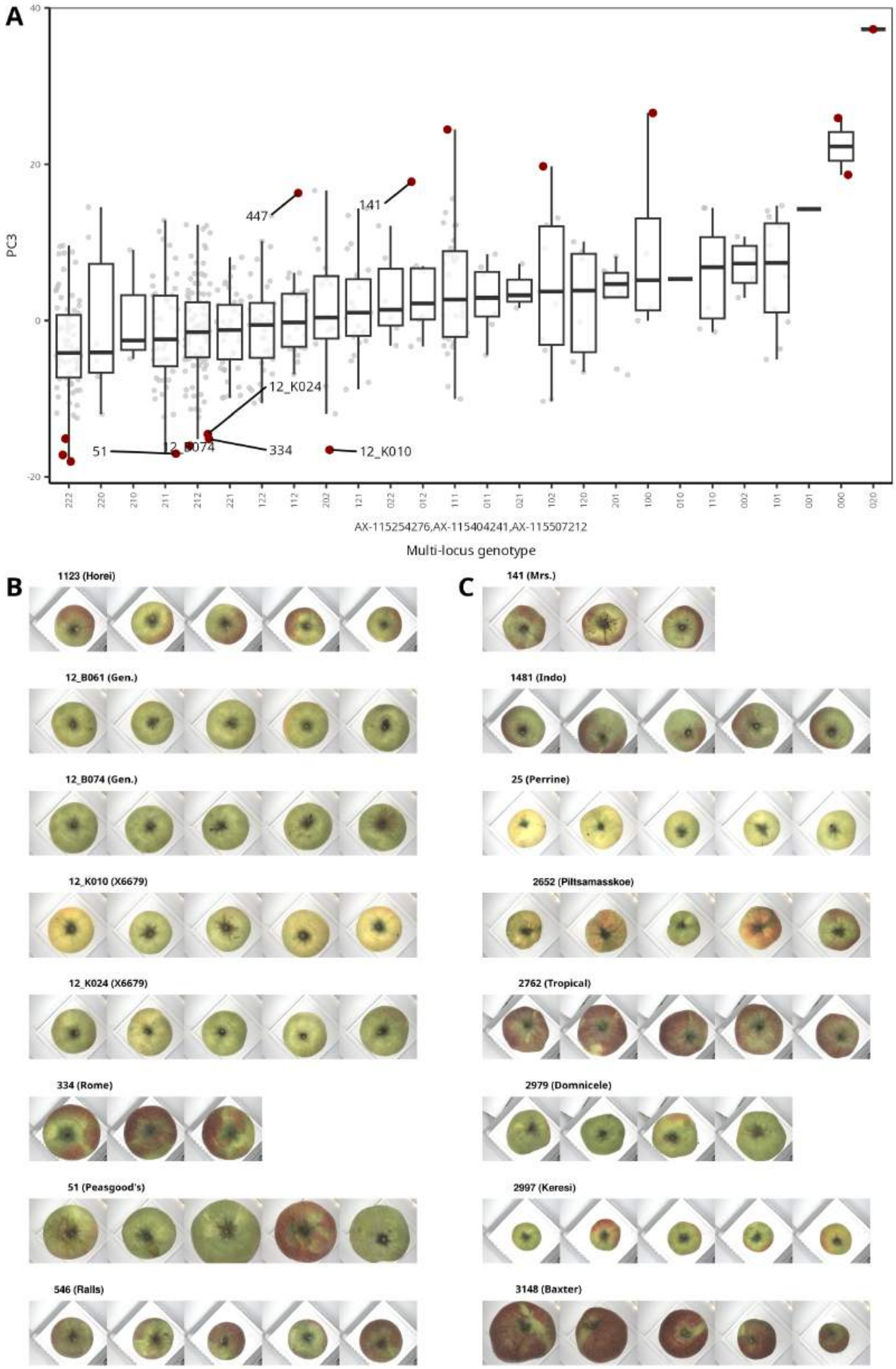

Figure S9: Combined effect of the SNPs identified for principal component 3 (PC3) on the shape of apples. The SNPs were assembled into multi-locus genotypes. A: Boxplots of the effect of each multi-locus genotype on PC3. The multi-locus genotypes are labeled using combinations of allele dosage (0, 1 or 2 alternative alleles) of the SNPs. The eight most extreme phenotypes for low and high phenotypic trait values are depicted in red. Their fruit images with up to five replicates are shown in B: and C:, respectively.

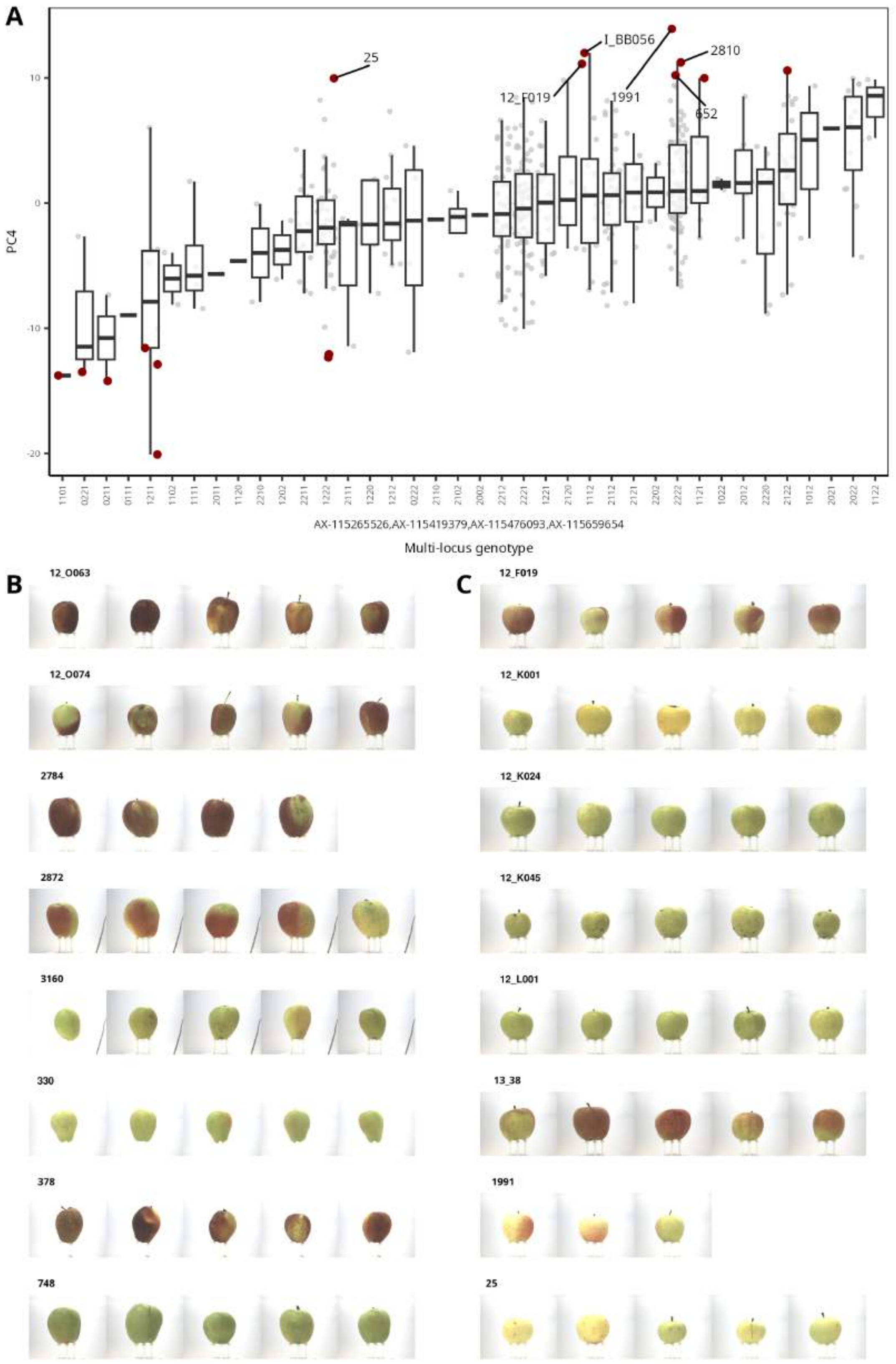

Figure S10: Combined effect of the SNPs identified for principal component 4 (PC4) on the shape of apples. The SNPs were assembled into multi-locus genotypes. A: Boxplots of the effect of each multi-locus genotype on PC4. The multi-locus genotypes are labeled using combinations of allele dosage (0, 1 or 2 alternative alleles) of the SNPs. The eight most extreme phenotypes for low and high phenotypic trait values are depicted in red. Their fruit images with up to five replicates are shown in B: and C:, respectively.

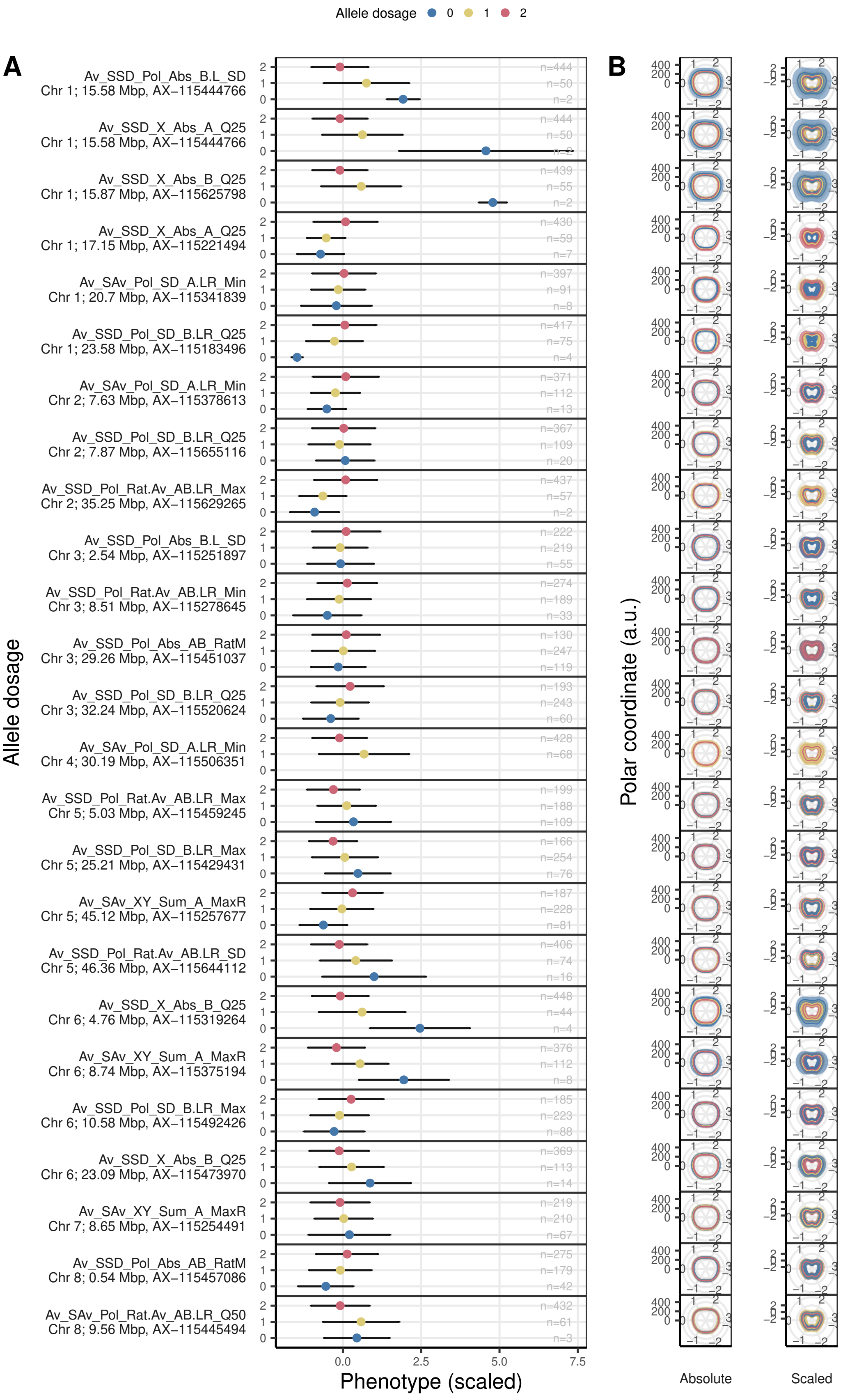

Figure continues on next page.

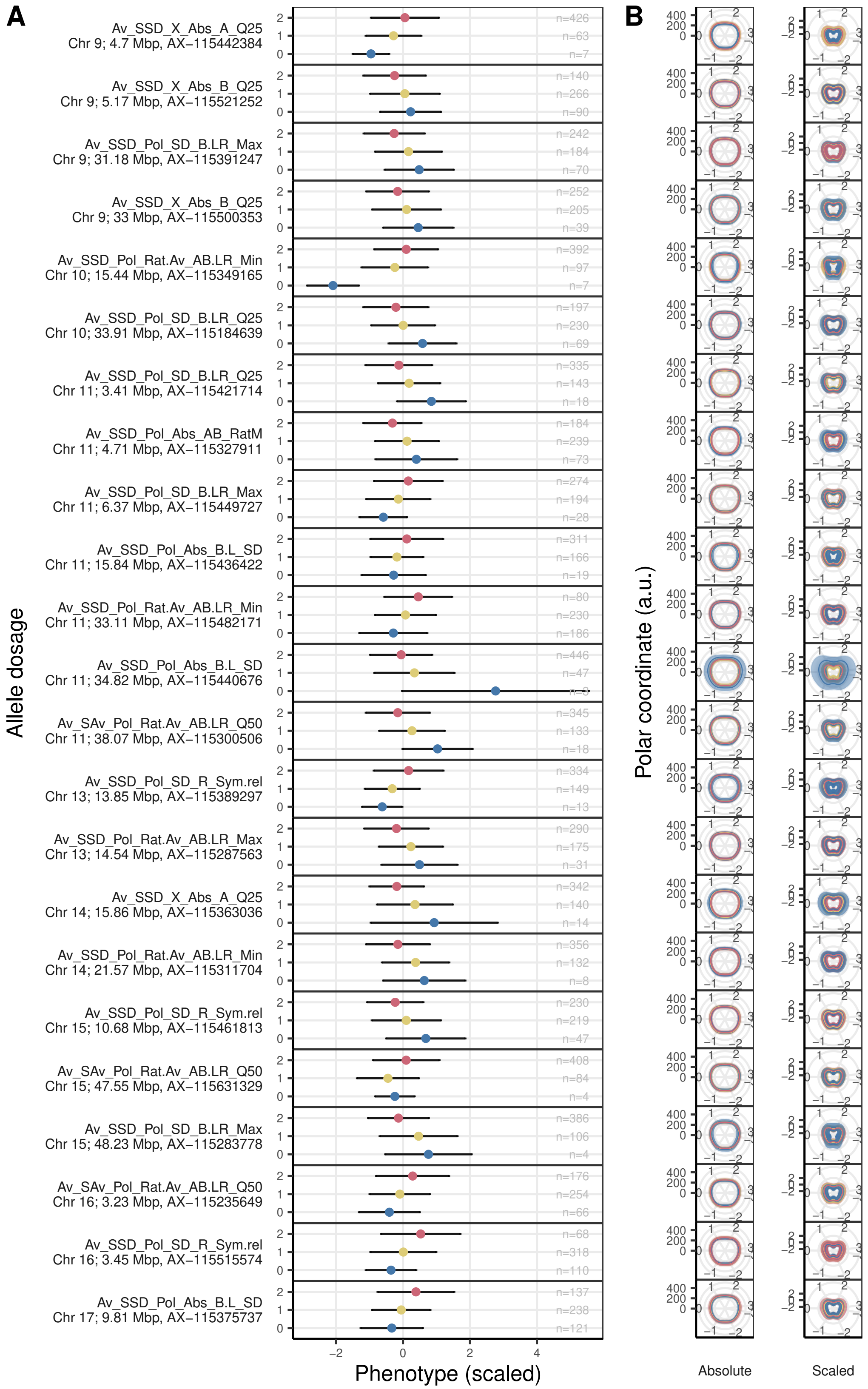

Figure S11: Effect of SNPs significantly associated with selected features (r<0.75) for fruit shape and size. A: Effect of the allele dosage (0, 1 or 2 alternative alleles) on the scaled values of PCs. B: The effect of each SNP on the averaged side-view fruit contour in absolute and relative values. The relative values were scaled (mean=0 and standard deviation=1) for every individual apple fruit with the resulting minimum value being the center of the graph, i.e., the visualized diameter size is arbitrary, and the shape effect is enhanced. The error bars show the standard deviation. See previous page to view the the full figure.

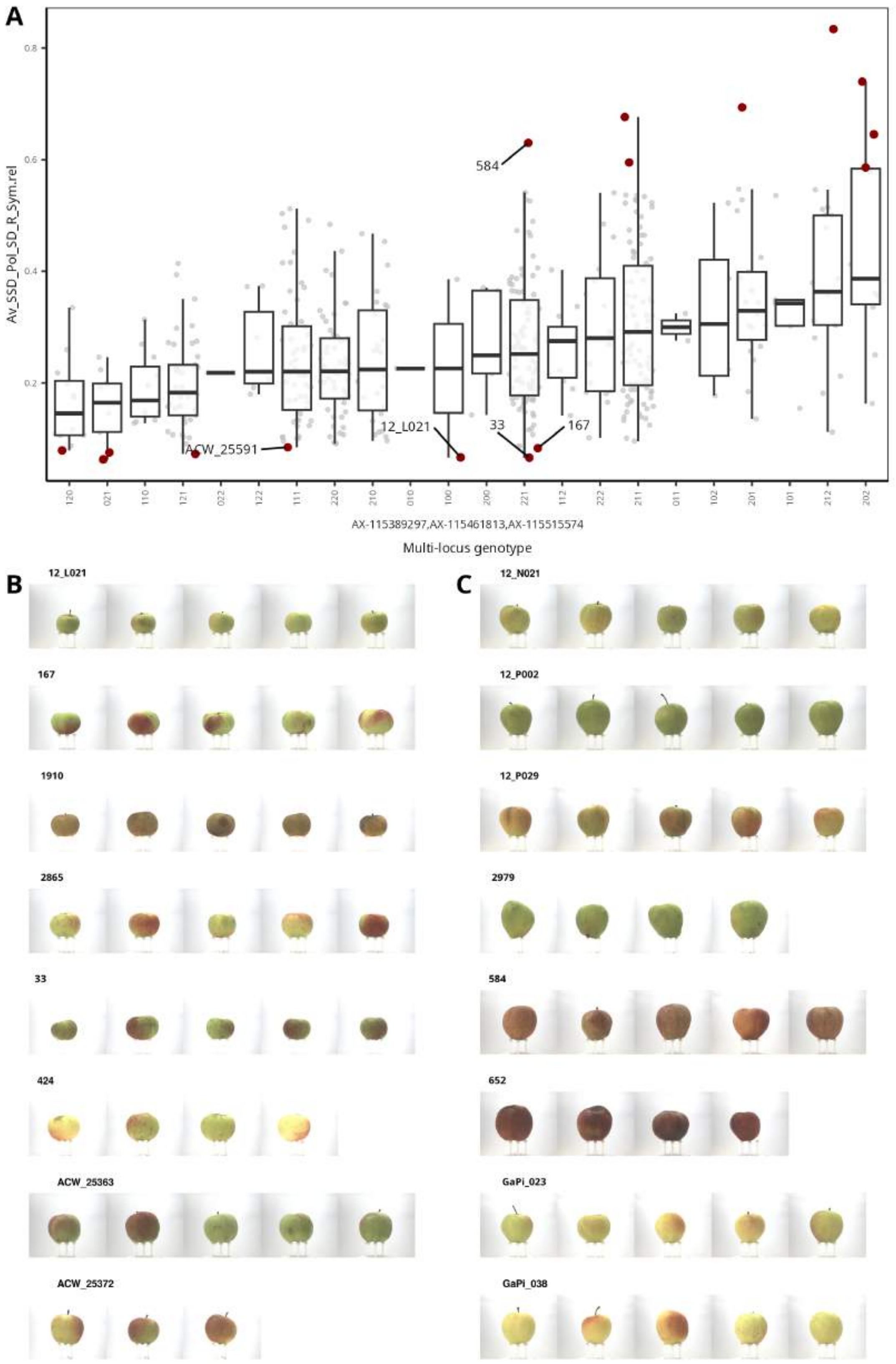

Figure S12: Effect of multi-locus genotypes build from SNPs AX-115389297, AX-115461813, and AX-115515574 that were significantly associated with the feature for symmetry Av_SSD_Pol_SD_R_Sym.rel of apple fruits. A: Boxplots of the multi-locus genotype effects on the symmetry feature. The multi-locus genotypes are labeled using combinations of allele dosage (0, 1 or 2 alternative alleles) of the SNPs. The eight most extreme phenotypes for low and high phenotypic trait values are depicted in red. Their fruit images with up to five replicates are shown in B: and C:, respectively.

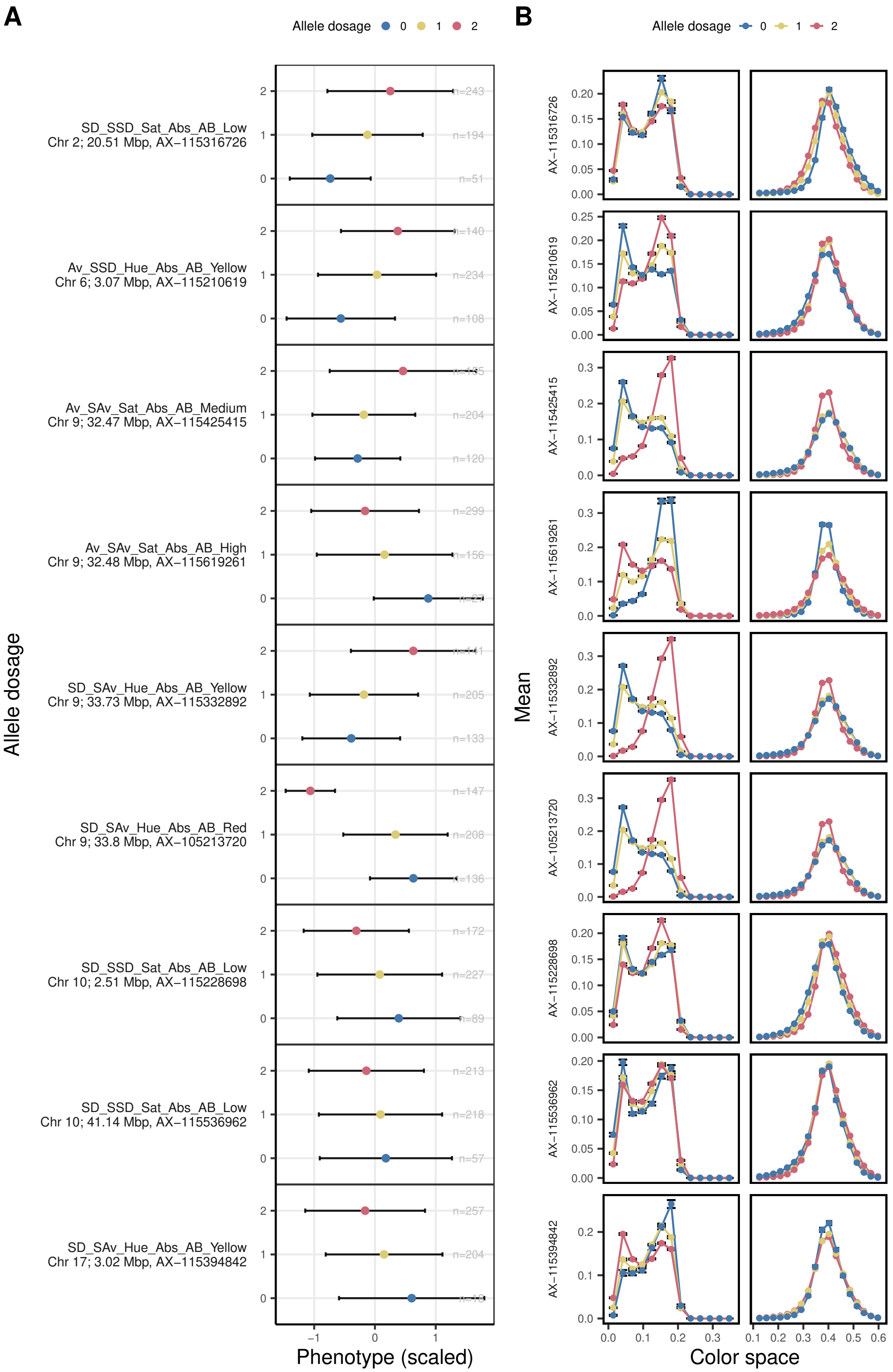

Figure S13: Allele dosage effect (0, 1 or 2 alternative alleles) on the hue and saturation color space for the selected color features. The error bars show the standard deviation of all genotypes per allele dosage. A: The position, the name of the SNP marker and the effect of the allele dosage is shown (0, 1 or 2 alternative alleles). The phenotypes were scaled for each feature to allow for a relative comparison. The error bar shows the standard deviation. B: The effect of SNPs on the hue and saturation color space. The error bars show the standard deviation of all genotypes per allele dosage. The Bonferroni-corrected significance level of the considered associations was 5%.

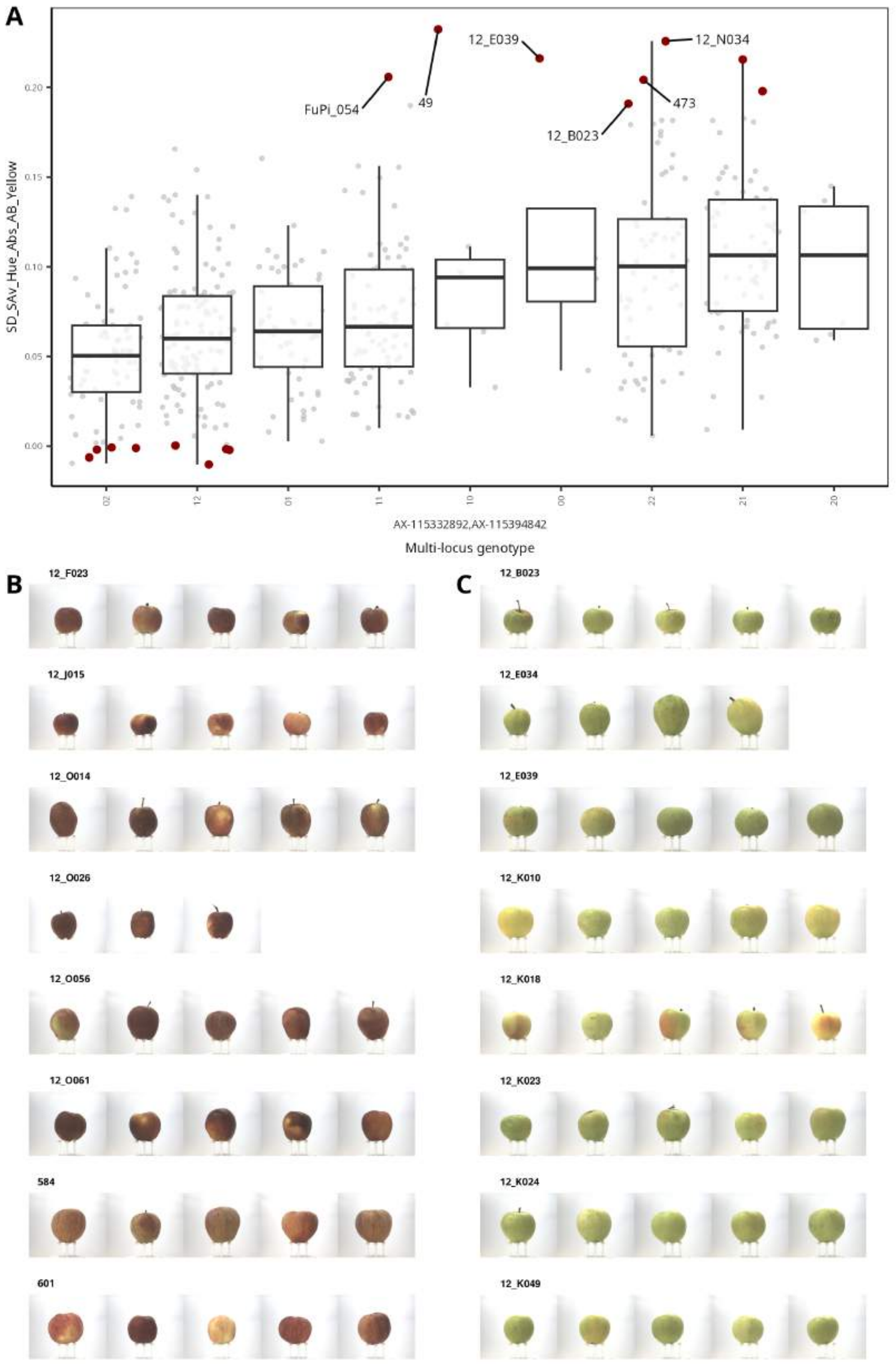

Figure S14: Effect of multi-locus genotypes build from SNPs that were significantly associated with the feature for color SD_SAv_Hue_Abs_AB_Yellow of apple fruits. A: Boxplots of the multi-locus genotype effects on the color feature. The multi-locus genotypes are labeled using combinations of allele dosage (0, 1 or 2 alternative alleles) of the SNPs. The eight most extreme phenotypes for low and high phenotypic trait values are depicted in red. Their fruit images with up to five replicates are shown in B: and C:, respectively.
